# Intracellular Adhesion Molecule‐1 Improves Responsiveness to Immune Checkpoint Inhibitor by Activating CD8^+^ T Cells

**DOI:** 10.1002/advs.202204378

**Published:** 2023-04-25

**Authors:** Se‐Hoon Lee, Yeongmin Kim, Bu‐Nam Jeon, Gihyeon Kim, Jinyoung Sohn, Youngmin Yoon, Sujeong Kim, Yunjae Kim, Hyemin Kim, Hongui Cha, Na‐Eun Lee, Hyunsuk Yang, Joo‐Yeon Chung, A‐Reum Jeong, Yun Yeon Kim, Sang Gyun Kim, Yeonhee Seo, Sehhoon Park, Hyun Ae Jung, Jong‐Mu Sun, Jin Seok Ahn, Myung‐Ju Ahn, Hansoo Park, Kyoung Wan Yoon

**Affiliations:** ^1^ Division of Hematology‐Oncology, Department of Medicine, Samsung Medical Center Sungkyunkwan University School of Medicine Seoul 06351 South Korea; ^2^ Department of Health Sciences and Technology, Samsung Advanced Institute of Health Sciences and Technology Sungkyunkwan University Seoul 06351 South Korea; ^3^ Department of Biomedical Science and Engineering Gwangju Institute of Science and Technology (GIST) Gwangju 61005 South Korea; ^4^ Genome and Company Pangyo‐ro 253, Bundang‐gu. Seoungnam‐si Gyeonggi‐do 13486 South Korea; ^5^ Division of Nephrology, Department of Medicine, Chosun University Hospital Chosun University School of Medicine Gwangju 61452 South Korea; ^6^ Medical Research Institute Sungkyunkwan University School of Medicine Seoul 06351 South Korea; ^7^ NEX‐I Inc. Seoul 05854 South Korea

**Keywords:** anti‐tumor effects, CD8^+^ T cells, chemokine (CXC motif) ligand 13, immune checkpoint inhibitor, soluble ICAM‐1

## Abstract

Immune checkpoint inhibitor (ICI) clinically benefits cancer treatment. However, the ICI responses are only achieved in a subset of patients, and the underlying mechanisms of the limited response remain unclear. 160 patients with non‐small cell lung cancer treated with anti‐programmed cell death protein‐1 (anti‐PD‐1) or anti‐programmed death ligand‐1 (anti‐PD‐L1) are analyzed to understand the early determinants of response to ICI. It is observed that high levels of intracellular adhesion molecule‐1 (ICAM‐1) in tumors and plasma of patients are associated with prolonged survival. Further reverse translational studies using murine syngeneic tumor models reveal that soluble ICAM‐1 (sICAM‐1) is a key molecule that increases the efficacy of anti‐PD‐1 via activation of cytotoxic T cells. Moreover, chemokine (CXC motif) ligand 13 (CXCL13) in tumors and plasma is correlated with the level of ICAM‐1 and ICI efficacy, suggesting that CXCL13 might be involved in the ICAM‐1‐mediated anti‐tumor pathway. Using sICAM‐1 alone and in combination with anti‐PD‐1 enhances anti‐tumor efficacy in anti‐PD‐1‐responsive tumors in murine models. Notably, combinatorial therapy with sICAM‐1 and anti‐PD‐1 converts anti‐PD‐1‐resistant tumors to responsive ones in a preclinical study. These findings provide a new immunotherapeutic strategy for treating cancers using ICAM‐1.

## Graphical abstract

Soluble intracellular adhesion molecule‐1 (sICAM‐1) is a co‐stimulatory molecule that activates CD4^+^ T cells and cytotoxic CD8^+^ T cells by interacting with lymphocyte function‐associated antigen‐1. sICAM‐1 induces anti‐tumor responses, reinvigorating the efficacy of anti‐programmed cell death protein‐1 (anti‐PD‐1) in cancers not responding to the anti‐PD‐1 single treatment. It suggests a therapeutic potential of sICAM‐1 in treating cancers.

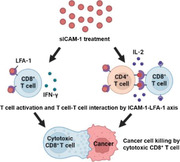

## 1 Introduction

T cell activation begins when T cell receptors detect foreign antigens. Ligand‐receptor interactions, including the immunoglobulin (Ig) superfamily and B7 family, affect this, stimulating or inhibiting a T cell‐mediated immune response.^[^ ^1^, ^2^, ^3^ ^]^ The most representative case is the PD‐1/PD‐L1 axis. Programmed cell death protein‐1 (PD‐1) is generally expressed on natural killer T cells, CD4^+^ T cells, CD8^+^ T cells, and B cells.^[^ ^4^, ^5^ ^]^ When PD‐1 interacts with its programmed death ligand‐1 (PD‐L1), PD‐1 induces an inhibitory signal that prevents CD28‐mediated T cell activation.^[^ ^5^, ^6^ ^]^ PD‐1 blockade, a monoclonal PD‐1 antibody, stimulates the overall immune responses in the tumor microenvironment (TME), decreasing tumor size.^[^ ^7^, ^8^ ^]^ PD‐1 blockade can target various cancers, including gastrointestinal cancer, lung cancer, and lymphomas, restoring T cell function and resulting in cancer regression.^[^ ^8^, ^9^, ^10^, ^11^ ^]^ However, in recent years, patients resistant to anti‐PD‐1 have emerged; there have been many attempts to uncover the mechanism of anti‐PD‐1 resistance, predict anti‐PD‐1 responders, and optimize patients' treatment schedules.^[^ ^12^, ^13^, ^14^, ^15^ ^]^ Clinical features such as tumor mutational burden, neoantigens, and immune infiltrates are the leading causes of resistance to anti‐PD‐1;^[^ ^16^, ^17^, ^18^, ^19^ ^]^ however, there are clear limits.^[^ ^15^, ^18^ ^]^ The reactivation of effector T cells is essential to overcoming the resistance to anti‐PD‐1.

Intracellular adhesion molecule‐1 (ICAM‐1) contributes to tumor removal by activating T cells with cell adhesion and co‐stimulatory functions.^[^ ^20^, ^21^, ^22^ ^]^ Lymphocyte function‐associated antigen‐1 (LFA‐1) expressed on T cells, a receptor for ICAM‐1, is crucial to this function via interaction with ICAM‐1 on antigen‐presenting cells.^[^ ^20^, ^22^, ^23^ ^]^ In addition, binding of LFA‐1 on cytotoxic T lymphocytes to ICAM‐1 is critical for releasing cytolytic granules into tumor cells by T cell activation.^[^ ^24^ ^]^ Many signaling pathways induce ICAM‐1 expression, including the Janus kinase (JAK)‐ signal transducer and activator of transcription (STAT) and tumor necrosis factor (TNF)‐related pathway.^[^ ^25^, ^26^ ^]^ Among these, chemokine (CXC motif) ligand 13 (CXCL13) is a key regulator of immune responses;^[^ ^27^, ^28^ ^]^ also known as B lymphocyte chemoattractant, CXCL13 interacts with CXC chemokine receptor type 5 (CXCR5) and elicits chemotactic effects.^[^ ^29^, ^30^ ^]^ CXCL13 regulates B cell organization in lymphoid follicles^[^ ^31^ ^]^ and is expressed by cells in peritoneal and pleural cavities, macrophages, and cancer cells. In addition, CXCL13 is a novel activator of ICAM‐1.^[^ ^32^ ^]^ CXCL13 induces vascular endothelial growth factor C (VEGFC), which promotes ICAM‐1.^[^ ^32^ ^]^

This study observed that ICAM‐1 expression was higher in the anti‐PD‐1‐responsive mouse cancer models. A loss of function study targeting ICAM‐1 revealed that it is a crucial molecule that determines the anti‐tumor efficacy of anti‐PD‐1 therapy. Interestingly, patients with non‐small cell lung cancer (NSCLC) responsive to immune checkpoint inhibitor (ICI) therapy have higher soluble ICAM‐1 (sICAM‐1) levels in their plasma. Recombinant sICAM‐1 potentiated T cell proliferation and cytokine production, but only when T cells were stimulated with T cell receptor (TCR). Moreover, CXCL13 and ICAM‐1 expression correlated. Treatment with anti‐PD‐1 and sICAM‐1 enhanced the anti‐tumor effects in anti‐PD‐1‐sensitive and ‐resistant mouse cancer models, indicating that ICAM‐1 restored anti‐PD‐1 efficacy. These results suggest that ICAM‐1 may be a novel molecule for cancer treatment.

## 2 Results

### 2.1 ICAM‐1 Up‐Regulation Led to a Higher Survival Rate and Immune Activation

**Figure 1:**
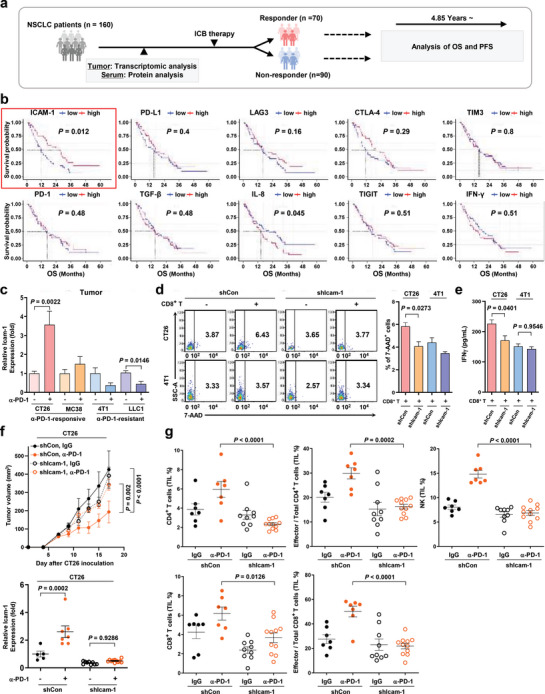
ICAM‐1 and clinical outcome in patients with NSCLC treated with ICI. a) Scheme for the human clinical study. A total of 161 patients with non‐small cell lung cancer (NSCLC) were assessed. The patients were divided into responders (*n* = 70), non‐responders (*n* = 90), and missed cases (*n* = 1) according to the RECIST 1.1 criteria: ICI, immune checkpoint inhibitor. b) OS of patients with NSCLC (*n* = 84) according to ICAM‐1 expression (cut‐off = median). A log‐rank test was used for statistical analysis. c) Quantitative PCR (qPCR) analysis of ICAM‐1 RNA levels in CT26, MC38, 4T1, and LLC1 cells before and after anti‐PD‐1 treatment. A two‐tailed unpaired *t*‐test was used for statistical analysis. Data are presented as mean ± s.e.m. (*n* = 4–10 mice per group). d) Quantification of CD8^+^ T cell cytotoxicity using flow cytometry analysis in CT26 and 4T1 treated with shIcam‐1 or shCon. One‐way ANOVA with Tukey's correction was used for statistical analysis. Data are presented mean ± s.e.m. (*n* = 3 per group). e) IFN‐*γ* production assessment using ELISA in the supernatant of the reaction mixture for cytotoxicity measurement. One‐way ANOVA with Tukey's correction was used for statistical analysis. Data are expressed as mean ± s.e.m. (*n* = 3 mice per group). f) shCon‐ or shIcam‐1‐CT26 tumor growth in mice treated with anti‐PD‐1 (*n* = 7–10 mice per group). Two‐way ANOVA with Tukey's correction was used for statistical analysis. Data are presented as mean ± s.e.m. qPCR analysis of ICAM‐1 was performed on RNA isolated from the mice used in immune profiling. One‐way ANOVA with Tukey's correction was used for statistical analysis. Data are expressed as mean ± s.e.m. (*n* = 5–11 mice per group). g) Quantification of T or NK cells among total lymphocytes in splenocytes or TILs from mice bearing shCon‐ or shIcam‐1‐CT26 tumors treated with anti‐PD‐1. The immune cell population was measured using flow cytometry. One‐way ANOVA with Tukey's correction was used for statistical analysis. Data are presented as mean ± s.e.m. (*n* = 7–11 mice per group).

We carried out gene expression profiles of tumor tissues from 160 NSCLC patients to evaluate the early determining factors of ICI response (Tables S1–S4, Supporting Information). After ICI therapy, the clinical outcomes, including overall survival (OS) and progression‐free survival (PFS), of ICI‐treated patients with NSCLC for 4.85 years, were documented (**Figure** **1**a). OS and PFS were significantly increased in patients with NSCLC and high ICAM‐1 expression (Figure 1b and Figure S1a,b, Supporting Information). However, no significant differences existed in OS and PFS between patients with high and low expression of other immune checkpoint proteins or the B7 family (Figure 1b and Figure S1a,b, Supporting Information). TGF‐*β* and interleukin 8 (IL‐8) are response‐predicting biomarkers of anti‐PD‐L1‐treated metastatic urothelial cancer (mUC), and high TGF‐*β* or IL‐8 reduces OS in patients with mUC treated with anti‐PD‐L1 therapy.^[^ ^33^, ^34^, ^35^ ^]^

Several studies have reported that interferon‐*γ* (IFN‐*γ*) signaling also drives beneficial clinical responses to ICI in melanoma.^[^ ^36^ ^]^ We observed the clinical outcomes of ICI‐treated patients with NSCLC for 4.85 years and assert that neither TGF‐*β* nor IFN‐*γ* impacts the clinical outcome of patients with NSCLC treated with ICI (Figure 1b). Based on 4.85‐year survival analysis, high level of ICAM‐1 was significantly correlated with the high survival probability of ICI‐treated NSCLC patients.

As ICAM‐1 levels correlated with ICI therapy's positive outcomes in patients, we investigated whether ICAM‐1 affects ICI‐responsive or ‐resistant phenotypes using several syngeneic mouse tumor models. First, we evaluated four syngeneic tumor models' phenotypes, including MC38, CT26, 4T1, and LLC1, which were responsive or resistant to anti‐PD‐1. After anti‐PD‐1 administration, the tumor size was reduced in MC38‐ and CT26‐bearing mice than in the control group (Figure S2, Supporting Information). In contrast, tumor size was unaffected in 4T1‐ or LLC1‐bearing mice. Here, we defined MC38 and CT26 as anti‐PD‐1‐responsive mouse tumor models, while 4T1 and LLC1 were defined as anti‐PD‐1‐resistant mouse tumor models. Next, ICAM‐1 levels in tumor tissues of anti‐PD‐1‐responsive and ‐resistant mouse models were analyzed. ICAM‐1 expression in MC38 and CT26 cells increased after treatment with anti‐PD‐1 but decreased in 4T1 and LLC1 cells compared to the control group (Figure 1c). To confirm the role of ICAM‐1 in immune modulation, ICAM‐1 was depleted in CT26 and 4T1 cancer cells using short hairpin RNA (shRNA) targeting ICAM‐1 (shIcam‐1) (Figure S3a, Supporting Information). First, we examined whether ICAM‐1 in cancer cells might regulate the anti‐tumor activity of CD8^+^ T cells. A cytotoxicity assay of cancer cells co‐cultured with CD8^+^ T cells revealed that ICAM‐1 depletion diminished CD8^+^ T cell‐mediated CT26 cell killing. Correlated with the level of ICAM‐1, 4T1 cancer cells were less lysed by CD8^+^ T cells than CT26 cancer cells, indicating that ICAM‐1 depletion in 4T1 rarely affects CD8^+^ T cell cytotoxicity (Figure 1d). The activity of CD8^+^ T cells was evaluated by measuring IFN‐*γ* release; IFN‐*γ* production in CD8^+^ T cells was significantly abated in CT26 cells than in 4T1 cells after ICAM‐1 depletion, suggesting that ICAM‐1 in CT26 cancer cells positively regulates CD8^+^ T cell activation (Figure 1e). Next, we explored the in vivo role of ICAM‐1 in an anti‐PD‐1‐treated mouse cancer model. The CT26 tumor volume upon anti‐PD‐1 treatment was diminished by ICAM‐1 depletion (Figure 1f). The downregulation of ICAM‐1 did not affect the proliferation of CT26 and 4T1 cancer cells (Figure S3b, Supporting Information). ICAM‐1 expression in tumor tissues was elevated in wild‐type CT26 treated with anti‐PD‐1 compared to the control group; however, no significant change was observed in ICAM‐1‐depleted CT26 (Figure 1f). Given that ICAM‐1 expression in cancer cells modulates the activation of CD8^+^ T cells, tumor tissues' immunological status was analyzed. Treatment with anti‐PD‐1 induced an increase in the population and activation of CD4^+^ T and CD8^+^ T and natural killer (NK) cells in CT26 cancer tissues, an anti‐PD‐1‐responsive tumor. However, ICAM‐1 depletion in CT26 significantly abolished the immune reactive phenotype upon anti‐PD‐1 treatment, reducing CD4^+^ T, CD8^+^ T, and NK cells (Figure 1g). Together, these data indicate that ICAM‐1 in cancer cells contributes to the anti‐tumor efficacy of anti‐PD‐1 by activating CD4^+^ T, CD8^+^ T, and NK cells.

### 2.2 Soluble ICAM‐1 Induces T Cell‐Specific Immune Responses

**Figure 2:**
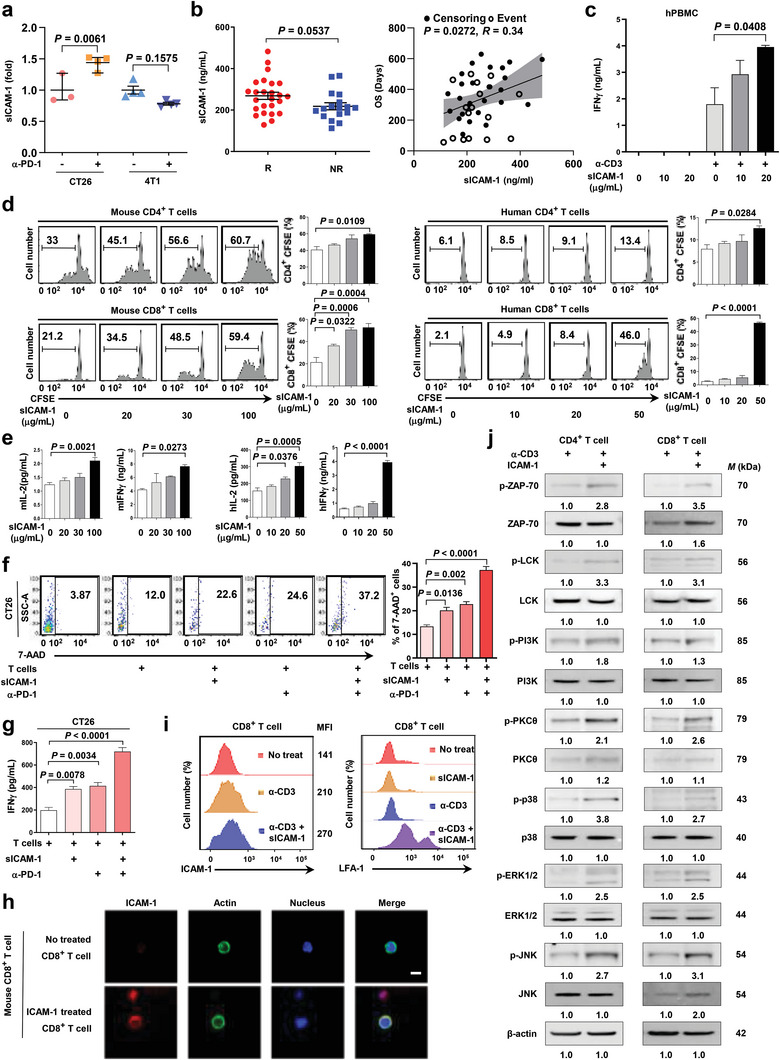
Activation of T cells via soluble ICAM‐1 in patients with NSCLC. a) sICAM‐1 production using ELISA in 4T1 and CT26 cancer cells treated or not treated with anti‐PD‐1. One‐way ANOVA with Tukey's correction was used for statistical analysis. Data are expressed as mean ± s.e.m. (*n* = 3–4 mice per group). b) sICAM‐1 production assessment using ELISA in human blood from anti‐PD‐1 (pembrolizumab) responders and non‐responders. A two‐tailed unpaired *t*‐test was used for statistical analysis. Data are presented as mean ± s.e.m. (*n* = 25 and 17 humans per group). The correlation between blood sICAM‐1 and OS of patients with NSCLC (censoring: *n* = 26, event: *n* = 16). *R* = 0.34. Simple linear regression was used for statistical analysis. c) IFN‐*γ* production using ELISA in human peripheral blood mononuclear cells (PBMC) treated with only human ICAM‐1 or human ICAM‐1 + anti‐CD3. One‐way ANOVA with Tukey's correction was used for statistical analysis. Data are expressed as mean ± s.e.m. (*n* = 3 humans per group). d) Representative flow cytometry analysis and quantification of Carboxyfluorescein succinimidyl ester (CFSE)‐stained T cells among total CD4^+^ or CD8^+^ T cells from mice and humans treated with sICAM‐1 in the presence of anti‐CD3. One‐way ANOVA with Tukey's correction was used for statistical analysis. Data are expressed mean ± s.e.m. (*n* = 3 mice or humans per group). e) IL‐2 production in CD4^+^ T cells or IFN‐*γ* production in CD8^+^ T cells from mice and humans in (d) using ELISA. One‐way ANOVA with Tukey's correction was used for statistical analysis. Data are presented as mean ± s.e.m. (*n* = 3 mice or humans per group). f) Quantification of CD8^+^ T cell cytotoxicity using flow cytometry of 7'AAD^+^ CT26 cells. Groups are classified into non‐treated, only splenocytes, splenocytes + sICAM‐1, splenocytes + mouse anti‐PD‐1, or splenocytes + mouse anti‐PD‐1 + sICAM‐1‐treated cells. One‐way ANOVA with Tukey's correction was used for statistical analysis. Data are expressed as mean ± s.e.m. (*n* = 3 per group). g) The supernatant of the reaction mixture for cytotoxicity measurement was obtained, and an ELISA was used to measure IFN‐*γ* production. One‐way ANOVA with Tukey's correction was used for statistical analysis. Data are presented as mean ± s.e.m. (*n* = 3 mice per group) h) Naïve mouse CD8^+^ T cells were treated with anti‐CD3 and sICAM‐1. Samples were analyzed using confocal microscopy (×400), which revealed the binding of ICAM‐1 on CD8^+^ T cells. Scale bar, 8 µm. i) ICAM‐1 and LFA‐1 expression on CD8^+^ T cells treated with anti‐CD3 or sICAM‐1. j) The expressions of total or phosphorylated T cell markers assessed using western blot analysis in CD4^+^ and CD8^+^ T cells treated with anti‐CD3; ICAM‐1; or the combination. *β*‐actin was used as a housekeeping protein.

ICAM‐1 induces T cell activation via association with LFA‐1.^[^ ^23^, ^37^ ^]^ We also confirmed the stimulatory effect of ICAM‐1 on CD4^+^ T or CD8^+^ T cells by incubating them with recombinant ICAM‐1 in a surface‐immobilized form with mouse or human CD4^+^ T or CD8^+^ T cells (Figures S4 and S5, Supporting Information). Next, we investigated whether CT26 and 4T1 cancer cells have different expression levels of ICAM‐1 on their cellular surfaces. Interestingly, we observed that the protein levels of ICAM‐1 on the surface of CT26 and 4T1 tumors were comparable (Figure S6, Supporting Information). ICAM‐1 has a soluble isoform and a membrane‐bound form; therefore, we evaluated soluble ICAM‐1 (sICAM‐1) production in cancer cells. We measured sICAM‐1 in the plasma of CT26 and 4T1 mouse tumor models that were untreated or treated with anti‐PD‐1. sICAM‐1 in blood plasma was increased in the CT26 model but not in the 4T1 model upon anti‐PD‐1 treatment, indicating that sICAM‐1 might predict an individual's response to anti‐PD‐1 (**Figure** **2**a). Consistently, sICAM‐1 in the plasma of patients with NSCLC also displayed higher levels in responders to ICI therapies than in non‐responders (Figure 2b and Tables S5–S7, Supporting Information). Moreover, higher levels of sICAM‐1 in the plasma of patients with NSCLC were highly correlated with better patient OS (Figure 2b).

sICAM‐1 is an effector of the response to anti‐PD‐1 therapy. We tested whether sICAM‐1 affects IFN‐*γ* production, known to activate immune cells, in PBMCs. sICAM‐1 did not affect IFN‐*γ* production in human PBMCs; nonetheless, it significantly induced IFN‐*γ* production when PBMCs were treated with anti‐CD3 (Figure 2c), suggesting that sICAM‐1 enhances the activation of PBMCs only when primary signals stimulate cells. We investigated whether sICAM‐1 regulates mouse and human T cell production. The proliferation of mouse and human CD4^+^ and CD8^+^ T cells was stimulated by sICAM‐1 in a dose‐dependent manner (Figure 2d). IL‐2 and IFN‐*γ* release from mouse and human CD4^+^ and CD8^+^ T cells also increased significantly (Figure 2e). The cytotoxic activity of CD8^+^ T cells toward CT26 cancer cells was increased by sICAM‐1 (10.6%: from 12.0% to 22.6%), comparable to anti‐PD‐1's extent of stimulation (12.6%: from 12.0% to 24.6%). Combining sICAM‐1 with anti‐PD‐1 enhanced the cancer cell‐killing activity of CD8^+^ T cells compared to treatment with sICAM‐1 or anti‐PD‐1 (25.2%: from 12.0% to 37.2%) (Figure 2f). Based on the increase in cytotoxicity compared to a single treatment, combining sICAM‐1 and anti‐PD‐1 had an additive effect, suggesting that sICAM‐1 and anti‐PD‐1 might independently activate T cells. Similarly, IFN‐*γ* production from CD8^+^ T cells co‐cultured with CT26 cancer cells was increased by sICAM‐1 and anti‐PD‐1 alone and additively enhanced by combining sICAM‐1 and anti‐PD‐1 (Figure 2g). sICAM‐1 did not affect the growth and viability of cancer cells, implying that sICAM‐1 treatment does not induce cancer cell aggressiveness (Figure S7, Supporting Information). Our confocal imaging analysis revealed that sICAM‐1 bound to the cellular surface of human and mouse CD4^+^ and CD8^+^ T cells when cells were activated by anti‐CD3 (Figure 2h and Figure S8, Supporting Information). ICAM‐1 and LFA‐1 expression on CD4^+^ and CD8^+^ T cells was significantly elevated by treatment with sICAM‐1 and anti‐CD3 (Figure 2i and Figure S9, Supporting Information). T cells with ICAM‐1 upregulation form synapse‐like structures with neighbor T cells. This homotypic interaction could induce cytokine production like IL‐2, activating another CD4^+^ and CD8^+^ T cells.^[^ ^38^, ^39^, ^40^ ^]^ We examined the cellular signaling pathways related to T cell activation. Anti‐CD3‐induced activation of phosphoinositide 3‐kinase (PI3K)/protein kinase B (AKT) and zeta‐chain‐associated protein kinase 70 (ZAP‐70) signaling, including phosphorylation of ZAP‐70, lymphocyte‐specific protein tyrosine kinase (LCK), PI3K, protein kinase C *θ* (PKC*θ*), extracellular signal‐regulated kinase 1/2 (ERK 1/2), p38, and c‐Jun N‐terminal kinase (JNK), was enhanced by sICAM‐1 (Figure 2j).

### 2.3 LFA‐1 Is Mainly Expressed on CD8^+^ TEM Cells

**Figure 3:**
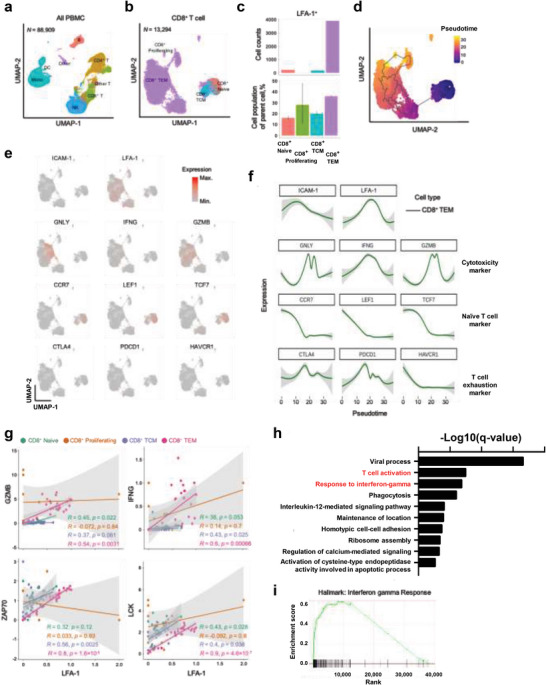
Single‐cell RNA sequencing of CD8^+^ T cells from patients with NSCLC. a) UMAP depicting the seven color‐coded immune populations. We identified CD8^+^ naïve T, CD8^+^‐proliferating T, CD8^+^ TEM, and CD8^+^ TCM cells. b) UMAP demonstrating the four color‐coded CD8^+^ T cell populations. c) Cell number and proportion of subphenotypes of CD8^+^ T cells expressing LFA‐1. d) Pseudotime trajectory analysis of CD8^+^ T cells based on the scRNA‐seq (Monocle3). e) UMAP exhibiting gene expression profiles of CD8^+^ T cells with functional annotation. f) The T cell marker genes plotted according to the CD8^+^ T cell trajectories. g) The correlation between the expression of marker genes and LFA‐1. Simple linear regression was used for the statistical analysis. h) Bar plot of pathways upregulated in LFA‐1‐high CD8^+^ TEM cells. i) Gene set enrichment analysis for IFN‐*γ* pathway in LFA‐1‐high CD8^+^ TEM cells.

To elucidate the role of ICAM‐1 in T cells, we analyzed the plasma of 38 patients with NSCLC and categorized the immune cells into seven clusters using single‐cell RNA sequencing (scRNA‐seq, **Figure** **3**a). The CD8^+^ T cells were subclustered into CD8^+^ naïve T, CD8^+^ proliferating T, CD8^+^ central memory T (TCM), and CD8^+^ effector memory T (TEM) cells (Figure 3b). LFA‐1 was predominantly expressed in the CD8^+^ TEM cluster but not in the other CD8^+^ T cell clusters (Figure 3c). Among the four CD8^+^ T cell subclusters, the TEM cluster was responsible for the cytotoxic function of the effector cells,^[^ ^41^ ^]^ which is the differentiated form of CD8^+^ naïve T cells, as revealed by the trajectory analysis (Figure 3d). LFA‐1 expression had the same tendency as the other cytotoxicity markers wherein LFA‐1, granulysin (GNLY), IFNG, and granzyme B (GZMB) were overexpressed in CD8^+^ TEM cells (Figure 3e,f). Consistent with this, LFA‐1's expression positively correlated with that of the T cell activation markers such as ZAP‐70 and LCK in addition to GZMB and IFNG (Figure 3g). CD8^+^ TEM cells were then classified into LFA‐1‐low or ‐high groups based on the level of LFA‐1 expression. Corresponding gene expression profiles were generated and analyzed. The LFA‐1‐high CD8^+^ TEM cells induced T cell activation and IFN‐*γ* pathways (Figure 3h). Thus, corroborating this observation, gene set enrichment analysis demonstrated a significant increase in IFN‐*γ* signaling in LFA‐1‐high CD8^+^ TEM cells (Figure 3i), indicating that ICAM‐1‐LFA‐1 axis is crucial to cytotoxic effector T cell function.

### 2.4 CXCL13 Is a Key Molecule That Regulates ICAM‐1 Expression

**Figure 4:**
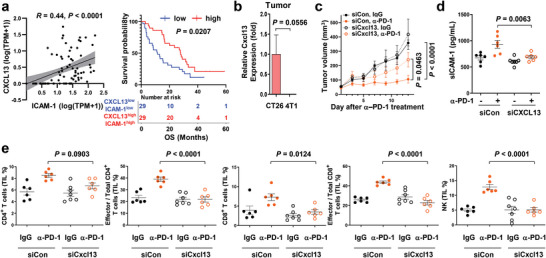
CXCL13 positively regulates ICAM‐1 expression and the efficacy of anti‐PD‐1 therapy. a) The correlation between the expression levels of ICAM‐1 and CXCL13 in patients with NSCLC (*n* = 84). *R* = 0.44. Simple linear regression was used for statistical analysis. OS in patients with NSCLC and low expression of ICAM‐1 and CXCL13 (*n* = 29) or vice versa (*n* = 29). A log‐rank test was used for statistical analysis. b) qPCR analysis of basal CXCL13 was performed on RNA isolated from CT26 and 4T1. A two‐tailed unpaired *t*‐test was used for statistical analysis. Data are expressed as mean ± s.e.m. (*n* = 3–4 mice per group). c) CT26 tumor growth in mice treated with anti‐PD‐1; siRNA‐targeting CXCL13; or the combination (*n* = 5 mice per group). Two‐way ANOVA with Tukey's correction was used for statistical analysis. Data are presented as mean ± s.e.m. d) sICAM‐1 production in blood from the mice used in immune profiling was assessed using ELISA. One‐way ANOVA with Tukey's correction was used for statistical analysis. Data are expressed as mean ± s.e.m. (*n* = 6–7 mice per group) e) Quantification of T cells or NK cells among total lymphocytes in splenocytes or TILs from mice bearing CT26 tumors treated with anti‐PD‐1; siCxcl13; or the combination using flow cytometry. One‐way ANOVA with Tukey's correction was used for statistical analysis. Data are expressed as mean ± s.e.m. (*n* = 6–7 mice per group).

Previous studies have revealed that sICAM‐1 level in human serum correlates with CXCL13. The CXCL13‐sICAM‐1 axis is a predictive value for clinical outcomes in inflammatory diseases.^[^ ^42^, ^43^, ^44^ ^]^ In our transcriptomic analysis of patients with NSCLC,

ICAM‐1 expression was correlated with CXCL13 expression (**Figure** **4**a). Moreover, patients with NSCLC and high CXCL13 and ICAM‐1 expression (CXCL13^high^; ICAM‐1^high^) had significantly higher OS than those with low CXCL13 and ICAM‐1 expression (CXCL13^low^; ICAM‐1^low^) (Figure 4a). Intratumoral CXCL13 levels were higher in the anti‐PD‐1‐responsive CT26 model than in the anti‐PD‐1‐resistant 4T1 model (Figure 4b). We depleted CXCL13 in a CT26 mouse tumor model to investigate whether CXCL13 contributes to tumor response to anti‐PD‐1 therapy. The tumor size reduction by anti‐PD‐1 treatment was significantly diminished by CXCL13 depletion (Figure 4c). In addition, CXCL13 depletion was confirmed in tumor tissues (Figure S10, Supporting Information). Intratumoral ICAM‐1 was decreased by CXCL13 depletion (Figure S10, Supporting Information). Consistently, anti‐PD‐1 treatment elevated plasma sICAM‐1 in the CT26 mouse tumor model, as previously observed. This increase in sICAM‐1 expression by anti‐PD‐1 was abrogated by CXCL13 depletion (Figure 4d). The immune cell status in tumor tissues was analyzed by measuring CD4^+^ T, CD8^+^ T, and NK cells of tumor‐infiltrating lymphocytes (TILs). Expansion of CD4^+^ T, CD8^+^ T, and NK cells and activation of CD4^+^ T and CD8^+^ T cells by anti‐PD‐1 treatment was significantly abrogated by CXCL13 depletion (Figure 4e), indicating that CXCL13 positively regulates sICAM‐1 and induces immune responses to anti‐PD‐1.

### 2.5 ICAM‐1 Enhances the Efficacy of Anti‐PD‐1 In Vivo

**Figure 5:**
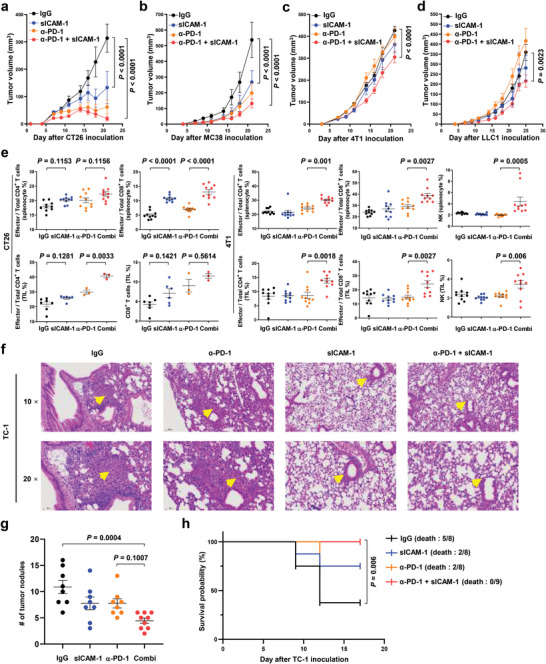
Anti‐tumor effects of combination treatment using soluble ICAM‐1 and anti‐PD‐1. a) CT26 and b) MC38 tumor growth in BALB/c or C57BL/6 mice (*n* = 7–10 mice per group) treated with sICAM‐1; anti‐PD‐1; or the combination. Two‐way ANOVA with Tukey's correction was used for statistical analysis. Data are expressed as mean ± s.e.m. c) 4T1 and d) LLC1 tumor growth in BALB/c or C57BL/6 (*n* = 6–8 mice per group) mice treated with sICAM‐1; anti‐PD‐1; or the combination. Two‐way ANOVA with Tukey's correction was used for statistical analysis. Data are presented as mean ± s.e.m. e) Quantification of T or NK cells among total lymphocytes in splenocytes or TILs from mice bearing CT26 or 4T1 tumors treated with sICAM‐1; anti‐PD‐1; or the combination using flow cytometry. One‐way ANOVA with Tukey's correction was used for statistical analysis. Data are expressed as mean ± s.e.m. (*n* = 3–10 mice per group). f–h) C57BL/6 mice were intratracheally injected with TC‐1 cancer cells for orthotopic model. f) The images of H&E‐stained orthotopic TC‐1 tumor tissues from C57BL/6 mice treated with sICAM‐1; anti‐PD‐1; or the combination at 10× or 20× magnifications. g) The number of tumor nodules in lungs from C57BL/6 mice treated with sICAM‐1; anti‐PD‐1; or the combination. One‐way ANOVA with Tukey's correction was used for statistical analysis. Data are presented as mean ± s.e.m. (*n* = 8–9 mice per group). h) The survival probability of C57BL/6 mice with orthotopic TC‐1 tumor treated with sICAM‐1; anti‐PD‐1; or the combination. A log‐rank test was used for statistical analysis. (*n* = 8–9 mice per group).

sICAM‐1 contributes to anti‐PD‐1 therapy response. Therefore, we evaluated whether sICAM‐1 has a combinational therapeutic effect with anti‐PD‐1 in anti‐PD‐1‐responsive syngeneic mice tumor models, CT26 and MC38. Tumor volume was significantly reduced when treated with sICAM‐1 or anti‐PD‐1 in CT26 and MC38 mouse tumor models (**Figure** **5**a,b). There was a synergistic therapeutic pattern of sICAM‐1 treatment combined with anti‐PD‐1 therapy in CT26 and MC38 tumors (Figure 5a,b). We applied sICAM‐1 treatment to anti‐PD‐resistant syngeneic tumor mouse models, 4T1 and LLC‐1. Tumor volume was not reduced when treated with anti‐PD‐1 or sICAM‐1 (Figure 5c,d). However, combining anti‐PD‐1 with sICAM‐1 reinvigorated the efficacy of anti‐PD‐1, resulting in decreased tumor size (Figure 5c,d). The tendency presented above was more evident in a spider plot of tumor growth (Figure S11, Supporting Information). Immune cell profiling of tumor tissues revealed that combining sICAM‐1 with anti‐PD‐1 led to the expansion and activation of CD4^+^ T and CD8^+^ T cells in CT26 tumor models (Figure 5e). The 4T1 tumor model displayed expansion and activation of CD4^+^ T, CD8^+^ T, and NK cells upon combination treatment with sICAM‐1 and anti‐PD‐1, compared to anti‐PD‐1 therapy alone (Figure 5e). We then investigated whether sICAM‐1 has anti‐tumor effects in orthotopic tumor models for physiologically similar TME. The survival probability of mice with orthotopic LLC1 tumors was significantly higher when treated with sICAM‐1 and anti‐PD‐1 compared to other groups (Figure S12, Supporting Information). Other lung cancer cell line, TC‐1, was injected intratracheally for orthotopic model. Hematoxylin and eosin (H&E) staining revealed that TC‐1 tumors were reduced upon sICAM‐1 treatment combined with anti‐PD‐1 therapy (Figure 5f and Figure S13, Supporting Information). The number of tumor nodules was also decreased when treated with sICAM‐1 and anti‐PD‐1 (Figure 5g). The survival probability had the same tendency as the H&E imaging and the number of tumor nodules (Figure 5h). To reveal the effects of sICAM‐1 on exhausted T cells, exhausted CD4^+^ and CD8^+^ T cells were first prepared using phytohemagglutinin‐L (PHA‐L) treatment. sICAM‐1 was added to treat the exhausted T cells to confirm the effect of sICAM‐1 on the exhausted T cells. No change in PD‐1 expression was observed even after sICAM‐1 treatment, suggesting that sICAM‐1 does not affect the exhausted T cells in vitro (Figure S14, Supporting Information). The ligand‐binding domain in LFA‐1 is only accessible to ICAM‐1 after T cell activation via TCR signaling.^[^ ^45^ ^]^ Therefore, LFA‐1 on exhausted T cells did not interact with sICAM‐1. Thus, our results indicate that CXCL13 can induce sICAM‐1, and sICAM‐1 enhances T cells' proliferation and their effector function and reduces tumor mass, suggesting the potential of sICAM‐1 as a new anti‐tumor therapy.

## 3 Discussion

Strategies for overcoming anti‐PD‐1 resistance have been studied. Various factors, including irreversible T cell exhaustion, oncogene mutations, dysfunction of major histocompatibility complex (MHC), tumor mutational burden, neoantigen, and immune infiltrate, have been suggested as etiologies of resistance.^[^ ^17^, ^18^, ^46^ ^]^ This situation can be solved by enhancing T cell priming, increasing T cell infiltration, or improving immunosuppressive TME.^[^ ^47^, ^48^, ^49^ ^]^ Immune checkpoint proteins are considered potent targets in cancer immunotherapy. According to their function in the TME and various immune cell subsets, these were classified into stimulatory or inhibitory immune checkpoints. Immune reinvigoration can be achieved by targeting inhibitory immune checkpoints. In recent years, various anti‐PD‐1, anti‐PD‐L1, and anti‐cytotoxic T lymphocyte antigen‐4 (anti‐CTLA‐4) have been used in clinical trials to treat patients with cancer.^[^ ^50^, ^51^ ^]^ Other immune checkpoints, including lymphocyte‐activation gene 3 (LAG3), T cell immunoglobulin and mucin domain‐containing 3 (TIM3), and T cell immunoreceptor with immunoglobulin and ITIM domain (TIGIT), emerged as new targets in patient‐specific ICI.^[^ ^52^, ^53^ ^]^ Regarding activators, target checkpoint proteins are rarely induced in the TME compared to normal tissues and can restore anti‐tumor effects by blocking immune evasion. Our research on patients with NSCLC suggests that high ICAM‐1 expression, not other checkpoints, is associated with high survival probability (Figure 1b). Also, ICAM‐1 expression, rather than PD‐1, is a predictive marker for anti‐PD‐1 therapy response.

ICAM‐1 expression is induced by proinflammatory cytokines in the tertiary lymphoid structure (TLS), an ectopic lymphoid formation containing B‐cell follicles and germinal centers in tumors.^[^ ^54^ ^]^ Intratumoral TLS formation is associated with high ICAM‐1 levels.^[^ ^21^ ^]^ ICAM‐1 is well‐known for its anti‐tumor effects by being involved in T cell priming by trans‐endothelial trafficking of effector T cells and facilitating immune cell adhesion with tumors.^[^ ^21^ ^]^ Thus, increased ICAM‐1 levels positively correlate with immune cell infiltration,^[^ ^21^ ^]^ suggesting the possibility of ICAM‐1 as a new immunotherapy. We discovered that sICAM‐1 stimulates antigen‐specific T cell activity. sICAM‐1 treatment enhanced the efficacy of anti‐PD‐1 by blocking tumor growth and increasing the infiltration of immune cells, such as effector T and NK cells. In particular, sICAM‐1 displayed more substantial anti‐tumor effects in the CT26 and MC38 anti‐PD‐1 responsive models due to their immunogenic characteristics.

ICAM‐1 appears to induce the transmigration of lymphocytes across vascular endothelia during inflammation. ICAM‐1 causes tyrosine phosphorylation and Ca^2+^ flux in cytotoxic T cells in the presence of anti‐CD3, which stimulates T cell activity.^[^ ^55^ ^]^ Several cytokines, including IL‐2 and IFN‐*α*, have been used as cancer therapies. Exogenous IL‐2 induces T cell‐mediated anti‐tumor cytotoxicity.^[^ ^56^ ^]^ IFN‐*α* has direct pro‐apoptotic activity on tumor cells and antiangiogenic activity, inhibiting tumor vasculature.^[^ ^57^ ^]^ However, these cytokine therapies have adverse side effects on the immune system. High‐dose IL‐2 can induce hypotension, renal failure, and fever.^[^ ^56^ ^]^ IL‐2 sometimes results in a cytokine storm, in which numerous inflammatory cytokines from NK and other immune cells are secreted abnormally.^[^ ^58^ ^]^ IFN‐*α* can also cause autoimmune diseases alone or when induced by cytokines, such as TNF‐*α* and IFN‐*γ*.^[^ ^59^ ^]^

Recently, the LFA‐1 agonist 7HP349 was in a clinical trial to treat patients with non‐immunogenic melanoma via combination treatment with CTLA‐4 blockade. It stimulates LFA‐1 on T cells, activating effector T cell functions. sICAM‐1 can also produce anti‐tumor effects similar to the LFA‐1 agonist. As mentioned above, sICAM‐1 selectively co‐stimulates the tumor‐specific T cells and promotes interaction among T cells, inducing the increase in effector T cells and differentiation into memory T cells later.^[^ ^38^, ^39^, ^45^ ^]^ In terms of drug safety, the toxicity of sICAM‐1 would be lower than that of small synthesized molecules, such as agonists, due to its biological properties, accompanying the minimal side effects to patients. Many clinical trials have also been conducted on T cell activation by other co‐stimulatory molecules as a combination strategy with anti‐PD‐1, as in the ICAM‐1/LFA‐1 axis.^[^ ^60^ ^]^ CD27 activates T cells and promotes effector and memory T cell formation when binding to CD70.^[^ ^61^ ^]^ Soluble CD70 enhances CD27‐mediated CD8^+^ T cell proliferation and production of IL‐2 and IFN‐*γ*.^[^ ^62^ ^]^ Varlilumab, a CD27 agonist, is combined with nivolumab in a clinical trial against ovarian and colorectal cancer.^[^ ^61^ ^]^ CD40/CD40L interaction upregulates pro‐inflammatory cytokines and ICAM‐1, activating macrophages and T cells, and its agonists are in clinical trials.^[^ ^61^, ^63^ ^]^ Other co‐stimulatory molecules, including 4‐1BB/4‐1BBL, OX40/OX40L, and glucocorticoid‐induced TNF receptor‐related protein (GITR)/GITR ligand (GITRL), have also been studied in clinical trials.^[^ ^61^ ^]^ Therefore, sICAM‐1 could be a promising immunotherapeutic candidate, with less non‐specific activation of T cells and minimal cytokine storms.

Our study has several limitations. First, the human clinical data focused only on NSCLC patients. Extending this study to other types of human cancers is crucial for a comprehensive understanding of ICAM‐1. Second, the efficacy of ICAM‐1 treatment was only confirmed in mouse models. Thus, ICAM‐1's side effects in the human body were not verified. Therefore, a clinical trial on patients with cancer is required. Finally, we revealed that anti‐PD‐1 (pembrolizumab) responders had higher sICAM‐1 levels than non‐responders only pre‐treatment (Figure 2b). Further post‐treatment studies are needed to prove the change in ICAM‐1 levels by anti‐PD‐1.

In summary, we observed that sICAM‐1 is associated with prolonged survival in patients with NSCLC and T cell activation in mouse models. Consistent with these data, we demonstrated that LFA‐1 is mainly expressed on CD8^+^ TEM cells, and cytotoxic markers conform to the tendency. We confirmed that CXCL13 regulates sICAM‐1 expression. In addition, our data revealed that sICAM‐1 treatment alone or in combination with anti‐PD‐1 is effective against tumors. In conclusion, our study suggests the therapeutic potential of sICAM‐1 for immune checkpoint protein therapy.

## 4 Experimental Section

### Human Sample Collection

All human clinical samples, including tumor tissue (*n* = 84) and blood (*n* = 77) from patients with NSCLC, were obtained with informed consent at the Samsung Medical Center. Sample collection was approved by the Institutional Review Board, Samsung Medical Center (2018‐04‐048, 2018‐06‐103, 2013‐10‐112).

### Mice and Cell Lines

BALB/c or C57BL/6 mice were purchased from Orient Bio (Gapyeong, Gyeonggi, Korea). All animal experiments were conducted following the Guide approved by the Institutional Animal Care and Use Committee of CHA University (190117). All animals used in this study were handled according to the policies approved by CHA University. CT26 (CRL‐2638, RRID:CVCL_7256), 4T1 (CRL‐2539, RRID:CVCL_0125), LLC1 (CRL‐1642, RRID:CVCL_4358) and Raw 264.7 (TIB‐71, RRID:CVCL_0493) cells were obtained from American Type Culture Collection (ATCC), and MC38 (ENH204‐FP, RRID:CVCL_B288) from Kerafast, which performs short tandem repeat DNA profiling for cell line authentication. TC‐1 cells were provided by Johns Hopkins University.

### Mouse cDNA and qPCR

Total RNA was extracted from mouse cancer tissues using RNeasy Mini Kit (QIAGEN) following the manufacturer's instructions. cDNA was prepared using the PrimeScript first strand cDNA synthesis kit (Takara). For measuring murine ICAM‐1 and CXCL13 expression at the RNA level, cDNA was analyzed via qPCR using TOPreal qPCR PreMIX (Enzynomics) and the following primers:

*β*‐actin: Sense 5′‐CGTGCGTGACATCAAAGAGAA‐3′
Antisense 5′‐TGGATGCCACAGGATTCCAT‐3′
ICAM‐1: Sense 5′‐CCGCAGGTCCAATTCACACT‐3′
Antisense 5′‐TGGATGCCACAGGATTCCAT‐3′
CXCL13: Sense 5′‐CATAGATCGGATTCAAGTTACGCC‐3′
Antisense 5′‐TCTTGGTCCAGATCACAACTTCA‐3′

### Protein Preparation

Simple cloning was performed to produce the ICAM‐1 protein. Mouse or human ICAM‐1 extracellular domain was cloned into a mouse (Invivogen, pfuse‐mchg1) or human IgG expression vector (Invivogen, pfuse‐hg40fc1). The plasmid was transfected into Expi293F cells using the ExpiFectamine 293 Transfection kit (Gibco, A14524). The protein was then purified from the supernatant using protein A beads (REPLIGEN, 10‐2500‐03) and an IgG Elution Buffer (Thermo Fisher Scientific, 21004).

### In Vitro Plate‐Bound T Cell Proliferation Assay

Regarding human T cells, blood samples were obtained from healthy donors with informed consent from the Gwangju Institute of Science and Technology (GIST). The study was approved by the institutional review board (20192009‐BR‐48‐02‐04). PBMCs were isolated using a Ficoll‐Paque Plus (GE Healthcare Life Sciences). CD4^+^ and CD8^+^ T cells were purified using a negative immunomagnetic selection kit (Miltenyi Biotec, Bergisch Gladbach, Germany). T cells (2 × 10^5^ per well) were activated with 2 µg mL^−1^ anti‐CD3 mAb (BioLegend, 100340) in the presence of ICAM‐1. Human sICAM‐1 treatment was at 0, 10, 20, and 50 µg mL^−1^. Spleens were collected from normal C57BL/6 mice for mouse T cells. Splenocytes were obtained by chopping the spleens. Isolation and stimulation of T cells are described above. Mouse sICAM‐1 treatment was at 0, 20, 30, and 100 µg mL^−1^. T cell proliferation was determined using CFSE (Invitrogen) dilution, measured using flow cytometry.

### ELISA Analysis

The concentrations of IL‐2 and interferon gamma (IFN‐*γ*) were measured in the supernatant via ELISA, using mouse IL‐2 (R&D systems, DY402), IFN‐*γ* ELISA kit (R&D systems, DY485), human IL‐2 (Invitrogen, 88‐7025‐88), and IFN‐*γ* ELISA kit (Invitrogen, 88‐7316‐88). The manufacturer's recommendations were followed. Thereafter, 96‐well plates (Corning Incorporated Life Sciences) were coated with 100 µL well^−1^ of IL‐2 or IFN‐*γ* capture antibody overnight and blocked with 100 µL well^−1^ of ELISA diluent in distilled water (D.W.) 100 µL of the samples were then applied to the wells in duplicates. For a standard curve, a recombinant standard was used at concentrations between 2000 and 31.25 *ρ*g mL^−1^ in mice and between 1000 and 15.625 *ρ*g mL^−1^ in humans. Streptavidin‐HRP conjugate was diluted to 1:40 in mice and 1:100 in humans in substrate solution. The reaction was stopped by adding 50 µL well^−1^ of 2 N H_2_SO_4_ (R&D systems, DY994), and the plates were analyzed at 450 nm using a SpectraMax Microplate Reader (Molecular Devices Corporation, Sunnyvale, USA). SoftMax Pro 7 software was used for analysis. Mouse ICAM‐1 (R&D systems, DY796) and human ICAM‐1 ELISA kit (R&D systems, DY720) were used to measure sICAM‐1 in mouse and human plasma. ELISA analysis was conducted using previously described protocols. Mouse plasma was collected on day 15 after CT26 and 4T1 tumor inoculation. The plasma of patients with NSCLC was collected from Samsung Medical Center (Tables S5–S7, Supporting Information).

### Cytotoxicity Assay

Splenocytes were isolated, as described previously. Isolated splenocytes were stimulated with anti‐CD28 and anti‐CD3 at 2 µg mL^−1^ concentration and cultured in complete DMEM medium (10% FBS) supplemented with 1% penicillin‐streptomycin and 50 U mL^−1^ IL‐2 (Roche, 11011456001) for 2 days. Then, CT26 or 4T1 cancer cells were stained with FITC dye (Thermo Fisher Scientific, C34554) for 30 min. Cells were washed, resuspended at 2 × 10^5^ cells/100 µL in complete medium, and plated in round‐bottom tubes. Splenocytes were added to the respective tubes at 10:1 effector: target (E: T) ratios for co‐culture. Each tube was treated with mouse sICAM‐1 or anti‐PD‐1 at 100 and 50 µg mL^−1^, respectively. The tubes were incubated at 37 °C for 6 h. After incubation, the cells were washed and stained with 7‐AAD dye (BioLegend, 420404). Data were acquired using CANTO II (BD Bioscience).

### T Cell Binding Assay

A 22 × 22 mm cover glass was placed on each well of a 6‐well plate. Anti‐CD3 (BioLegend, 100340, 10 µg mL^−1^) and anti‐CD28 (BioLegend, 102116, 2 µg mL^−1^) diluted in PBS coated each well and were incubated at 4 °C for 12 h. After suctioning the coating solution, isolated CD4^+^ or CD8^+^ T cells in complete RPMI supplemented with sodium pyruvate, L‐glutamine, and 2‐mercaptoethanol were placed in each well. T cells were treated with 50 µg mL^−1^ ICAM‐1 and incubated at 37 °C for 1–2 h in a 5% CO_2_ atmosphere. The secondary antibody was treated at 1:1000 dilution. Cells were observed using a confocal laser scanning microscope (Carl Zeiss, LSM 800) and analyzed using Zen 2.3 (blue edition, Carl Zeiss Microscopy GmbH, 2011). The secondary antibody information was as follows: Goat anti‐human IgG Alexa Fluor 568 (Thermo Fisher Scientific, A‐21090, RRID:AB_2535746), goat anti‐mouse IgG2a Alexa Fluor 568 (Thermo Fisher Scientific, A‐21134), DAPI solution (Abcam, ab228549), and phalloidin‐Fluor 488 reagent (Abcam, ab176753).

### Western Blotting

Isolated mouse CD4^+^ and CD8^+^ T cells were activated using anti‐CD3 and ICAM‐1. Cells were lysed in cell lysis buffer (Cell Signaling Technology, 9803S). Samples were heated with equal volumes of sodium dodecyl sulfate (SDS) sample buffer (Bio‐rad, 161–0737) with 2‐mercaptoethanol for 5 min at 95 °C and loaded onto 4–10% Tris‐glycine gels. Electrophoresis and transfer to nitrocellulose membranes (Bio‐Rad) were then performed. The membranes were blocked using Tris‐buffered saline (TBS) with 0.1% Tween 20 and 3% bovine serum albumin (BSA, Millipore, 82‐100‐6). Antibodies specific for phospho‐p44/42 MAPK (pERK1/2, cell signaling technology, 9101), p44/42 MAPK (ERK1/2, cell signaling technology, 9102), phospho‐PI3 kinase p85/p55 (cell signaling technology, 4228), PI3 kinase p85 (cell signaling technology, 4292), phospho‐Lck (cell signaling technology, 2751), Lck (cell signaling technology, 2752), phospho‐ZAP‐70/Syk (cell signaling technology, 2701), ZAP‐70 (cell signaling technology, 2705), phospho‐PKC*θ* (cell signaling technology, 9377), phospho‐p38 MAPK (cell signaling technology, 9211), p38 MAPK (cell signaling technology, 9212), phospho‐SAPK/JNK (cell signaling technology, 9251), SAPK/JNK (cell signaling technology, 9252), and *β*‐actin (Sigma, A2228) were used. Membranes were then treated with secondary antibodies and visualized using chemiluminescence (Thermo Fisher Scientific, 34577). The secondary antibody information was as follows: Anti‐mouse IgG HRP‐linked antibody (Cell Signaling Technology, 7076, RRID:AB_330924) and Anti‐rabbit IgG HRP‐linked antibody (Cell Signaling Technology, 7074, RRID:AB_2099233).

### Tumor Studies in Mice

Regarding tumor growth experiments, C57BL/6 mice were subcutaneously injected with 2 × 10^5^ MC38 or 5 × 10^5^ LLC1 cancer cells, and Balb/c mice with 1 × 10^6^ CT26 or 3 × 10^5^ 4T1 cancer cells per mouse, respectively. After tumor inoculation, tumor‐bearing mice were intraperitoneally injected with 10 mg kg^−1^ IgG (BioXCell, BE0089) or anti‐PD‐1 mAb (BioXCell, BE0146) on days 3, 7, 10, 14, 17, 20, and 10 mg kg^−1^ sICAM‐1 in PBS on days 2, 6, 9, 13, 18, and 19. Concerning the ICAM‐1‐deficient tumor model, BALB/c mice were subcutaneously injected with 1 × 10^6^ CT26 cells treated with control shRNA (shCon, Santa Cruz, sc‐108080) or shRNA targeting ICAM‐1 (shICAM‐1, Santa Cruz, sc‐29355‐V); 5 mg kg^−1^ IgG or anti‐PD‐1 mAb was intraperitoneally administered on days 1, 4, and 8. For the CXCL13‐deficient tumor model, BALB/c mice were subcutaneously injected with 1 × 10^6^ CT26 cells. Tumor‐bearing mice were intraperitoneally injected with 5 mg kg^−1^ IgG or anti‐PD‐1 mAb on days 1, 4, and 9 and intratumorally injected with 10 µg of siRNA targeting CXCL13 (siCXCL13) in PBS on days 1, 5, and 10. Tumor size was measured thrice a week until the endpoint, and tumor volume was calculated as length × width^2^ × 0.5. C57BL/6 mice were intercostally injected with 5 × 10^5^ LLC1 cancer cells along the median axillary line in the left lung for the orthotopic mouse model. C57BL/6 mice were intratracheally injected with 5 × 10^5^ TC‐1 cancer cells for the orthotopic mouse model. Tumor‐bearing mice were intraperitoneally injected with 5 mg kg^−1^ IgG or anti‐PD‐1 mAb and 10 mg kg^−1^ sICAM‐1 in PBS twice weekly. The number of tumor nodules in lung was measured after euthanizing the mice. The tumor tissue was analyzed using hematoxylin and eosin (H&E) staining. The tumor tissue was fixed using 10% formalin and embedded in paraffin for sectioning. The sections were stained with H&E and observed using microscope at 10× and 20× magnifications.

### Exhausted T Cell Production

Isolated CD4^+^ and CD8^+^ T cells were incubated for 5 days in complete media with 10 µg mL^−1^ PHA‐L (Merck, 11249738001). IL‐2 treatment was at 50 U mL^−1^ every 2 days. sICAM‐1 treatment was at 100 µg mL^−1^ on day 7, and flow cytometry was performed on day 9. T cell exhaustion was evaluated by measuring PD‐1 expression. The antibody information was as follows: PE rat IgG2b *κ* isotype (Biolegend, 400608) and anti‐mouse PD‐1 (Biolegend, 109104).

### Flow Cytometry

For immune profiling, tumors and spleens were harvested on day 15 after tumor inoculation. Tumors were chopped and transferred to RPMI 1640 media (GIBCO) supplemented with 0.25 mg mL^−1^ hyaluronidase Type IV‐S, 50 µg mL^−1^ DNase type 1, 2.5 mg mL^−1^ collagenase type 1, 1.5 mg mL^−1^ collagenase type 2, and 1 mg mL^−1^ collagenase type 4. The samples were then incubated at 37 °C for 50 min and filtered using a 70‐µm cell strainer (BD Bioscience). Spleens were mashed in RPMI 1640 media, incubated in RBC lysis buffer (eBioscience), and filtered through a cell strainer. Splenocytes (1 × 10^6^) or tumor cells per well were treated with anti‐mouse CD16/CD32 (BD Bioscience) at 4 °C for 10 min to block the Fc receptor. Surfaces were stained, and the fixation/permeabilization buffer set (BioLegend) solution was added. Intracellular staining was then performed. Stained cells were acquired using CANTO II (BD Bioscience), and data were analyzed using FlowJo software (TreeStar, RRID:SCR_008520). The antibody information was as follows: Anti‐CD3 (Biolegend, 100218), anti‐CD45 (Biolegend, 103116, RRID:AB_312981), anti‐CD4 (Biolegend, 100422), anti‐CD8 (Biolegend, 100706), anti‐CD44 (Biolegend, 103008), anti‐CD62L (Biolegend, 104412), anti‐NK1.1 (Biolegend, 108708), anti‐CD335 (Biolegend, 137603), APC/Cy7 rat IgG2b *κ* isotype (Biolegend, 400624), PerCP/Cyanine5.5 rat IgG2b *κ* isotype (Biolegend, 400632), PE/Cy7 rat IgG2b *κ* isotype (Biolegend, 400618), FITC rat IgG2a *κ* isotype (Biolegend, 400506), PE rat IgG2b *κ* isotype (Biolegend, 400608), APC rat IgG2a *κ* isotype (Biolegend, 400512), PE mouse IgG2a *κ* isotype (Biolegend, 400212), and PE rat IgG2a *κ* isotype (Biolegend, 400508).

4 × 10^5^ MC38, CT26, 4T1, and LLC1 cells were dyed with anti‐ICAM‐1 (Biolegend, 116108) and PE rat IgG2b *κ* isotype (Biolegend, 400608) to confirm ICAM‐1 expression on cancer cells. Regarding T cells, CD4^+^ and CD8^+^ T cells were isolated and divided into three groups: no‐treat, only anti‐CD3, and anti‐CD3 + sICAM‐1. Each group was incubated for 3 days and dyed with anti‐ICAM‐1. Anti‐CD3 and sICAM‐1 treatments were at 2 and 100 µg mL^−1^, respectively. Data analysis was performed as described above.

2 × 10^5^ CD4^+^ and CD8^+^ T cells were stimulated with 2 µg mL^−1^ anti‐CD3 and 5 µg mL^−1^ ICAM‐1 to measure LFA‐1 expression on mouse T cells. Anti‐LFA‐1 (Biolegend, 141006) and PE rat IgG1 *κ* isotype (Biolegend, 400408) were used for flow cytometry.

### Transcriptome Analysis

Tumor tissues were obtained from patients’ lungs via biopsy, and RNA was extracted from tissues using the RNeasy Mini Kit (QIAGEN). Next, 151‐bp paired‐end libraries were constructed from 1 µg of RNA using the TruSeq RNA Sample Prep Kit v2 (Illumina). Whole‐transcriptome sequencing (WTS) was performed on an Illumina HiSeq instrument. RNA‐seq reads from each WTS experiment were aligned to the human reference genome (GRCh38) using STAR aligner.^[^ ^64^ ^]^ Gene expression was quantified using RSEM.^[^ ^65^ ^]^

### Library Preparation and Pre‐Processing of scRNA‐seq

The library was prepared using Chromium Next Gem Single cell 5′ kit v2 (10x Genomics, 1000263) and Chromium Single Cell Human TCR Amplification kit (10x Genomics, 1000252). Cell suspension pools were made in groups of four donors per 10x Chromium run for genetic demultiplexing. With an expected cell recovery target of 20 000 per channel, ≈40 000 PBMCs extracted from each cell suspension pool were loaded on the 10x Chromium controller. The libraries were processed following the manufacturer's instruction (10x Genomics) and sequenced using Novaseq 6000 system in which 100‐bp paired‐end sequencing yields 25 000 reads per cell for scRNA‐seq. The raw data was processed using CellRanger v3.1 set with default (https://support.10xgenomics.com/single‐cell‐gene‐expression/software/pipelines/latest/what‐is‐cell‐ranger). The reads were lined up to the human reference genome (version hg19).

Reads with the same cell barcode, UMIs, and genes were grouped to calculate each cell's number of UMIs per gene using the “count” command. The UMI count tables of each cellular barcode were used for further analysis. The matrix data from Cell Ranger were processed with the Seurat package (version 4.0.4) in R software (version 4.1.1)^[^ ^66^ ^]^ for each sample. Gene expression data from each sample were first processed using the Read 10X function and then the CreateSeuratObject function with metadata was run to prepare the Seurat object. Whole Seurat objects were integrated into one object to reduce the batch effect and perform post analyses. The cells were first filtered out from the merged Seurat object as the number of expressed genes: genes <200 and > 2500, and the percent of mitochondrial genes over 15% of total expressed genes. Doublets also were calculated using Scrublet and filtered out before post analysis.

### Data Integration and Clustering

The merged Seurat object, including quality‐checked cells, was split individually by subjects and normalized using the SCTransform function with the top 2000 highly variable genes. SelectIntegrationFeatures function was performed to obtain highly variable genes. Cell clustering and UMAP visualization were performed using the FindClusters and RunUMAP functions, respectively. The annotations of cell identity on each cluster were defined using reference data with the expression of known marker genes of PBMCs.^[^ ^66^ ^]^ Pseudotime trajectory analysis of single cells was generated using Monocle 3 package (version 1.0.0)^[^ ^67^ ^]^ in R. The new_cell_data_set function was applied to create an object with the default parameters. CD8^+^ T naïve cell was chosen for root principal point to determine the pseudotime for CD8^+^ T cell.

### Differentially Expressed Genes (DEGs) Identification and GO Enrichment Analysis

The differentially over‐expressed genes in the specific cluster were identified when compared to other cell clusters with the Wilcoxon Rank‐Sum Test with the FindMarkers function in Seurat and the differentially expressed genes (adjusted *P*‐value < 0.05 and log2(FC) > 1.5) were used to perform ClueGO to find a biological pathway. ClueGO is a tool, a plugged‐in CytoScape, combining GO terms to create functionally grouped annotations in a network.^[^ ^68^ ^]^ GO Biological Process database of *Homo sapiens* was used for functional enrichment analysis. Significantly enriched GO terms were determined using a two‐sided hypergeometric test with a Bonferroni correction (*P* < 0.05). The degree of connectivity between terms in the network was calculated using groups based on a kappa score greater than 0.4 with a network specificity of 4–10. The GSEA was also used with the curated gene sets to identify the induced or repressed pathways between the cell clusters. The R package AUCELL6^[^ ^69^ ^]^ was used to estimate gene set scores per cell to identify DEG sets. For this analysis, the hallmark (referred to as “H”) and GO (“C5”) gene sets were used from the MSigDB^[^ ^70^ ^]^ v6.2 and exported using the R package GSEABase (v1.54.0).

### Statistical Analysis

Kaplan–Meier survival graphs were used for estimating the OS and PFS patterns. The censored points are indicated using cross marks. The log‐rank test was used for *P‐*values. Correlations were determined using Pearson's correlation method. Finally, statistical analyses were performed using the R‐3.6.0 program. Statistical significance was set at P < 0.05.

## Conflict of Interest

The authors declare no conflict of interest.

## Author Contributions

S.‐H.L., Ye.K., B.‐N.J., and G.K. contributed equally to this work. S.‐H.L., H.K., H.C., N.‐E.L., S.P., H.A.J., J.‐M.S., J.S.A., and M.‐J.A. analyzed clinical data. S.‐H.L., Ye.K., G.K., B.‐N.J., and K.W.Y. wrote the manuscript. G.K. analyzed survival and scRNA‐seq data. Ye.K., B.‐N.J., and Y.Y. performed cytometry analysis. J.S., Y.Y.K., and S.G.K. performed syngeneic mouse model experiments. Y.S. performed orthotopic mouse experiments. A.‐R.J. performed gene cloning. S.K. and Yu.K. extracted cloned DNA. H.Y. and J.‐Y.C. performed protein production. H.P. and K.W.Y. designed and supervised all experiments and analyses.

## Supporting information

Supporting Information

Supporting Information

Supplemental Table 1

Supplemental Table 2

## Data Availability

The data that support the findings of this study are available from the corresponding author upon reasonable request.
