# Supplementary material for: Intracellular Adhesion Molecule‐1 Improves Responsiveness to Immune Checkpoint Inhibitor by Activating CD8+ T Cells
Source: Adv Sci (Weinh). 2023 Apr 25;10(17):2204378. doi: 10.1002/advs.202204378 (PMC10265102; doi:10.1002/advs.202204378)
Supplement: Supplementary file 1 — Supporting Information [file ADVS-10-2204378-s001.pdf]

ADVANCED SCIENCE
Open Access

Supporting Information

for *Adv. Sci.*, DOI 10.1002/advs.202204378

Intracellular Adhesion Molecule-1 Improves Responsiveness to Immune Checkpoint Inhibitor by Activating CD8$^+$ T Cells

Se-Hoon Lee, Yeongmin Kim, Bu-Nam Jeon, Gihyeon Kim, Jinyoung Sohn, Youngmin Yoon, Sujeong Kim, Yunjae Kim, Hyemin Kim, Hongui Cha, Na-Eun Lee, Hyunsuk Yang, Joo-Yeon Chung, A-Reum Jeong, Yun Yeon Kim, Sang Gyun Kim, Yeonhee Seo, Sehhoon Park, Hyun Ae Jung, Jong-Mu Sun, Jin Seok Ahn, Myung-Ju Ahn, Hansoo Park and Kyoung Wan Yoon**

Intracellular adhesion molecule-1 improves responsiveness to immune checkpoint inhibitor by activating CD8$^+$ T cells

Supplementary Figures and Legends : pages 2-16

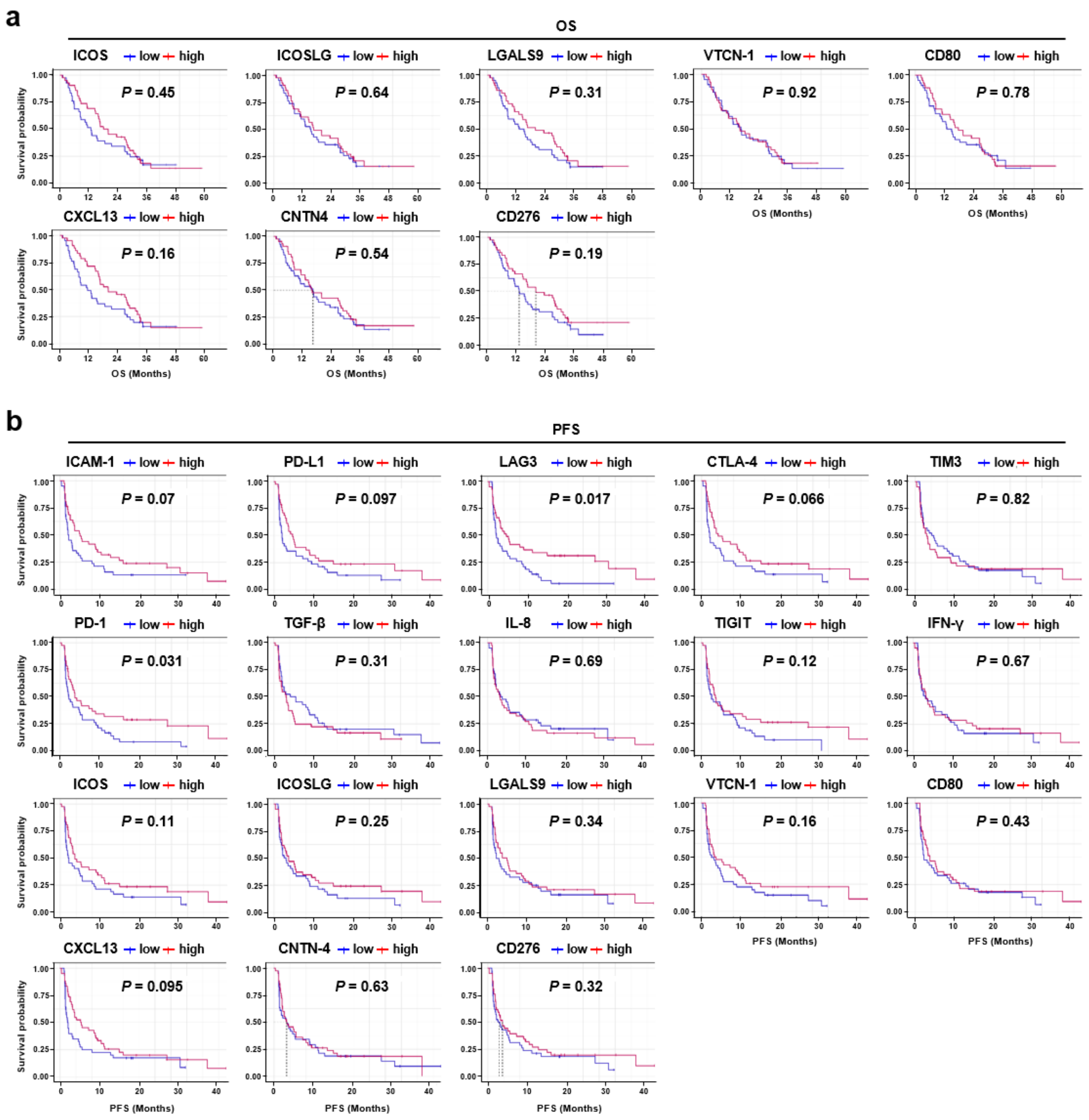

Supplementary Figure 1. Survival rates of NSCLC patients according to biomarker expression. (**a** and **b**) Overall survival (**a**) and progression-free survival (**b**) of NSCLC patients (*n* = 84) according to the expression levels of ICAM-1, PD-L1, LAG3, CTLA-4, TIM3, PD-1, TGF-β, IL-8, TIGIT, IFN-γ, ICOS, ICOSLG, LGALS9, VTCN-1, CD80, CXCL13, CNTN-4, and CD276. The log-rank test was used for the statistical analysis.

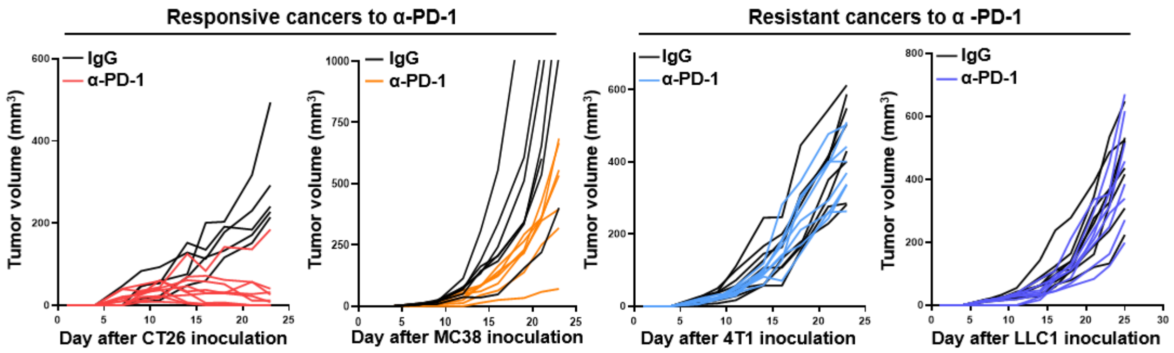

Supplementary Figure 2. Anti-PD-1 response in cancer cells. CT26, MC38, 4T1, and LLC1 tumor growth in mice treated with anti-PD-1 (*n* = 5–10 mice per group).

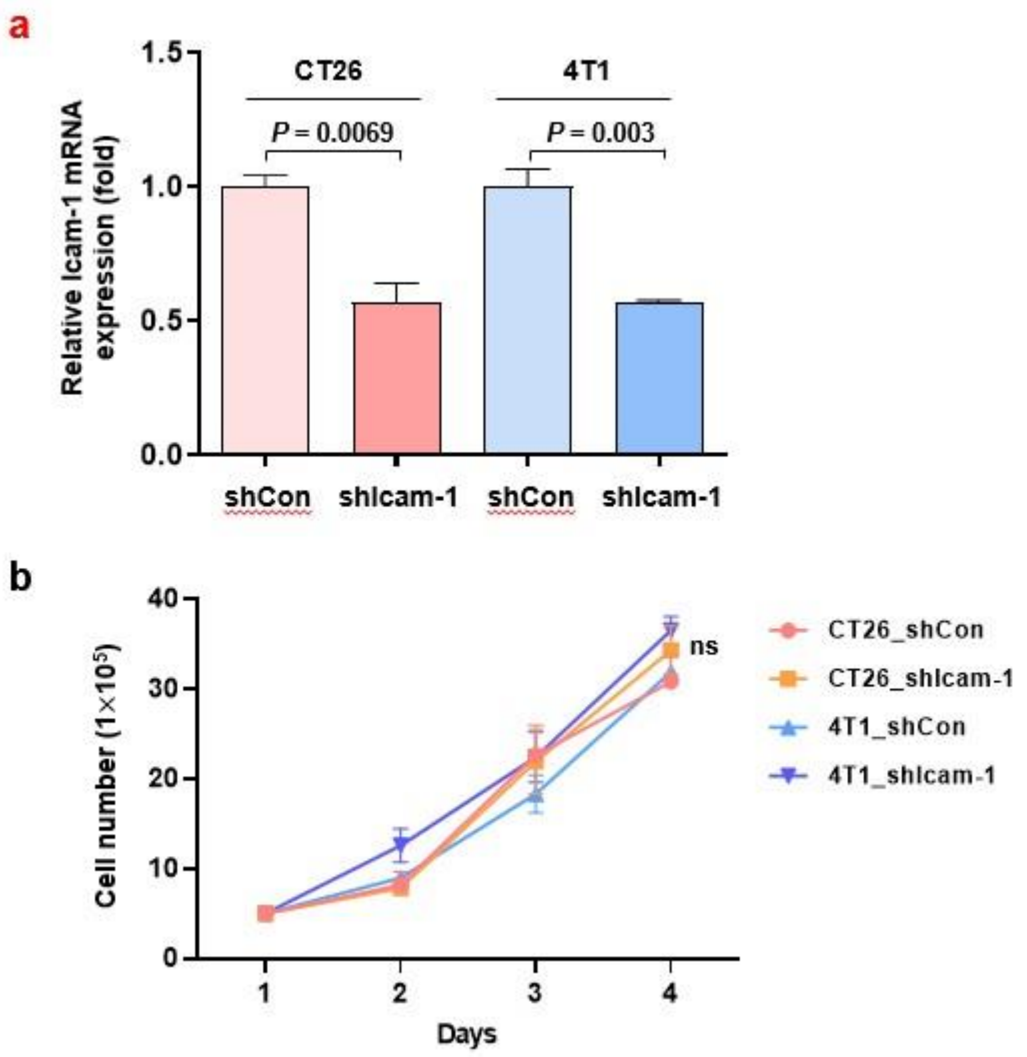

Supplementary Figure 3. Effect of *Icam-1* downregulation on cancer cells. a *Icam-1* expression on CT26 and 4T1 cancer cells treated with shCon or shIcam-1 using qPCR. One-way ANOVA with Tukey's correction was used for statistical analysis. Data are presented as mean ± s.e.m. (n = 3 per group). **b** Proliferation of CT26 and 4T1 cancer cells treated with shCon or shIcam-1 are shown. Data are presented as mean ± s.d.

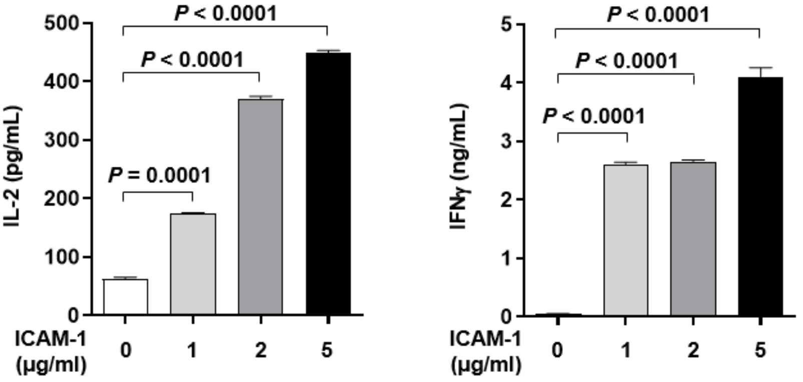

Supplementary Figure 4. Secretion of cytokines by bound ICAM-1 in mice. IL-2 or IFN-γ production via ELISA in $CD4^+$ and $CD8^+$ T cells treated with ICAM-1. One-way ANOVA with Tukey's correction was used for statistical analysis. Data are presented as mean ± s.e.m. (*n* = 2 mice per group).

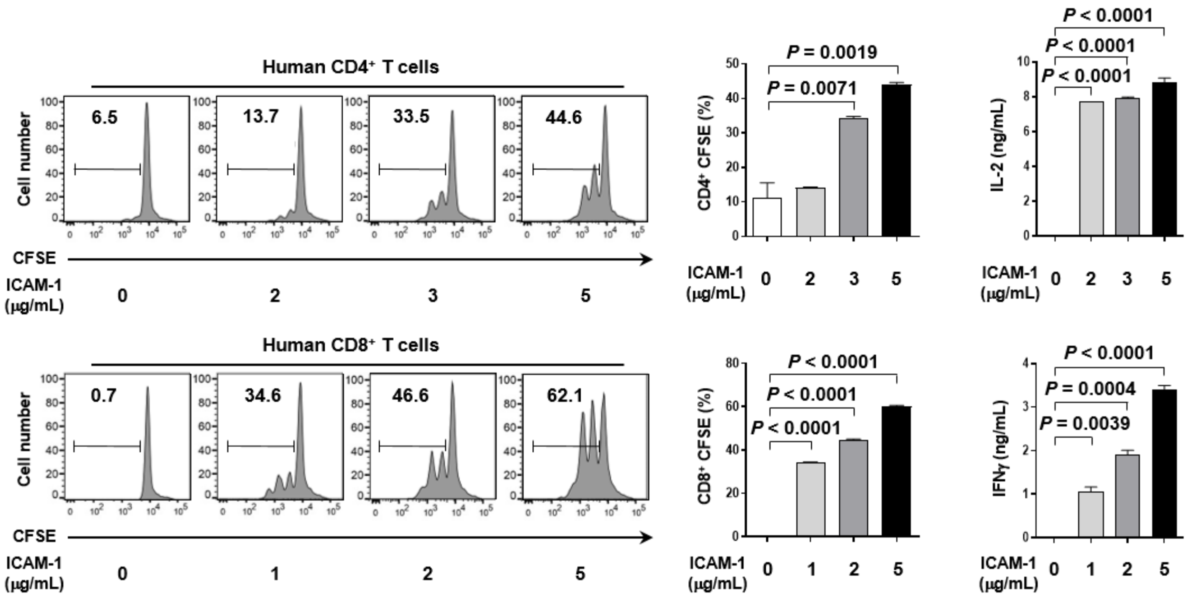

Supplementary Figure 5. Activation of T cells by bound ICAM-1 in humans. Representative flow cytometry analysis and quantification of CFSE-stained T cells among total $CD4^+$ or $CD8^+$ T cells. 2 μg/mL human anti-CD3 antibody and ICAM-1 at the indicated concentrations was treated. ELISA was performed to measure IL-2 production in $CD4^+$ T cells and IFN-γ production in $CD8^+$ T cells. One-way ANOVA with Tukey's correction was used for statistical analysis. Data are presented as mean ± s.e.m. (n = 2 humans per group).

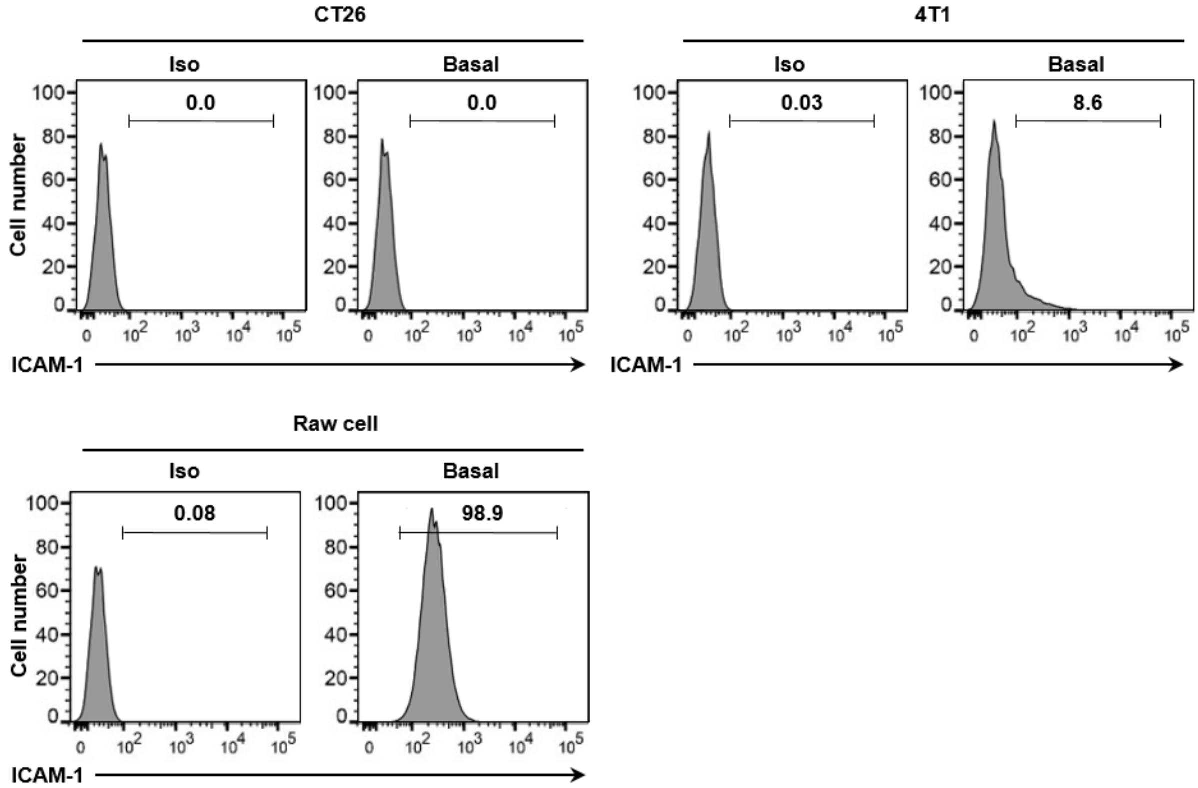

Supplementary Figure 6. ICAM-1 expression on the surface of mouse cancer cells. ICAM-1 expression on CT26, 4T1, and raw cells was measured by flow cytometry. Raw cells were used as positive controls.

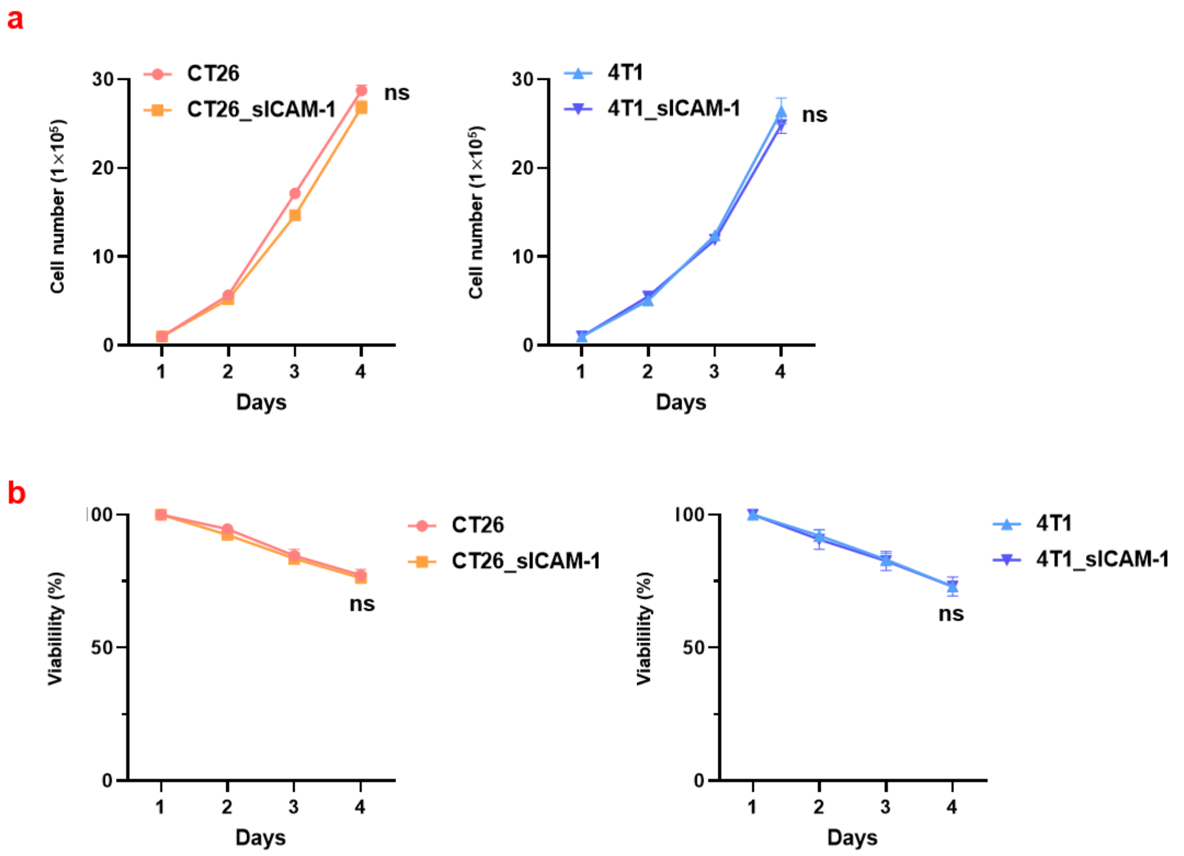

Supplementary Figure 7. The effect of sICAM-1 treatment on cancer cells. (**a** and **b**) CT26 and 4T1 cancer cells were treated or not treated with sICAM-1. The proliferation (**a**) and viability (**b**) of CT26 and 4T1 cancer cells treated with sICAM-1 or not is shown. Serum-free medium was used for culture to observe the effects of sICAM-1 on cancer cell viability in **b**. Data are presented as mean ± s.d. (n = 3 per group). One-way ANOVA with Tukey's correction was used to analyze Day 4 values.

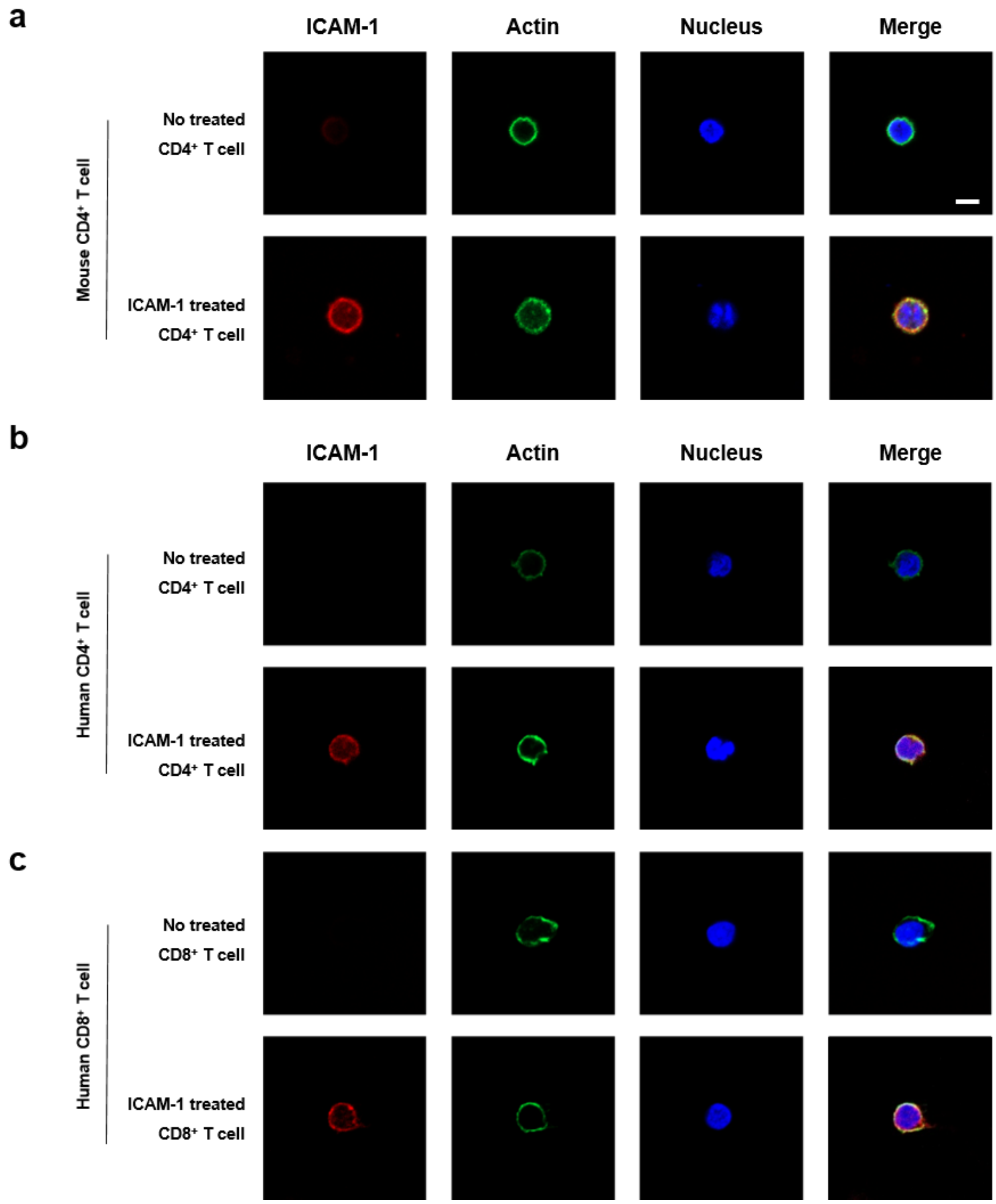

Supplementary Figure 8. Confocal microscopy showing the binding of ICAM-1 to $CD4^+$ and $CD8^+$ T cells. a Naïve mouse $CD4^+$ T cells were treated with anti-CD3, and sICAM-1. Anti-ICAM-1 was then treated, and samples were analyzed via confocal microscopy (×400), which showed the binding of ICAM-1 on $CD4^+$ T cells. (**b** and **c**) Naïve human $CD4^+$ and $CD8^+$ T cells treated with anti-CD3 and sICAM-1. Anti-ICAM-1 was used to detect the binding of

ICAM-1 to (**b**) $CD4^+$ and (**c**) $CD8^+$ T cells. Actin and nuclei were also stained as controls. Scale bar, 8 μm.

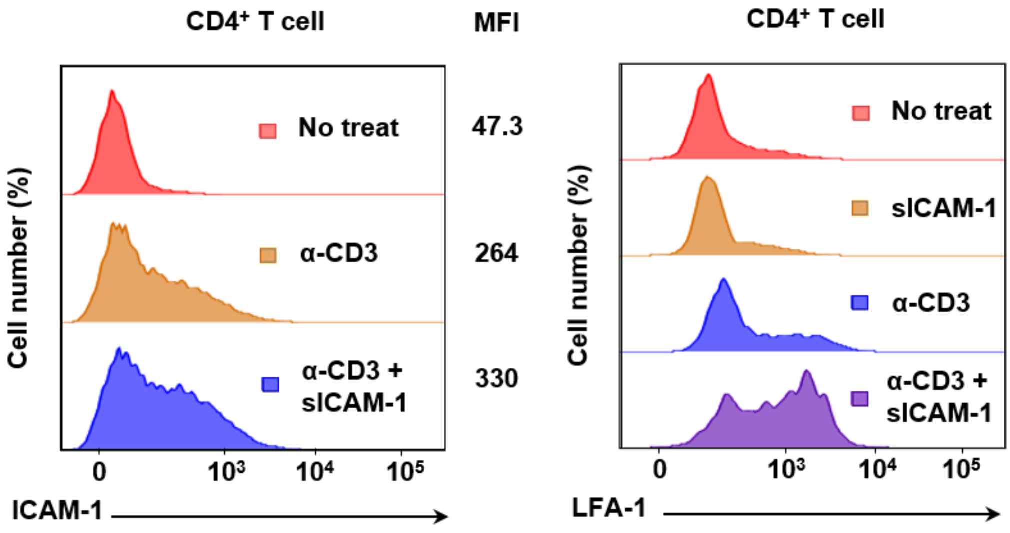

Supplementary Figure 9. The expression of ICAM-1 and LFA-1 on activated T cells. ICAM-1 and LFA-1 expression on CD4$^+$ T cells treated with anti-CD3 or sICAM-1 by flow cytometry analysis.

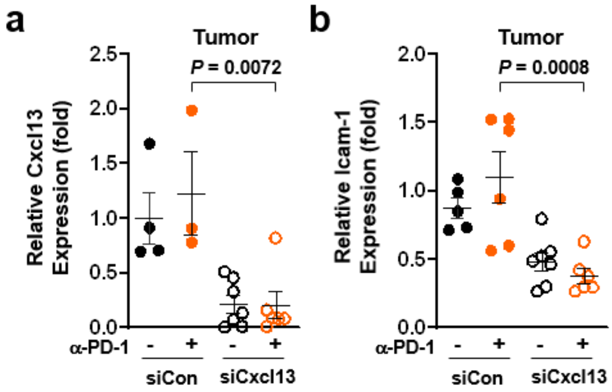

Supplementary Figure 10. RNA expression of *Cxcl13* and *Icam-1* in cancer cells with downregulation of *Cxcl13*. (**a** and **b**) *Cxcl13* (**a**) and *Icam-1* (**b**) expression on CT26 tumors from mice treated with siCon or siCxcl13 using qPCR. One-way ANOVA with Tukey's correction was used for statistical analysis. Data are presented as mean ± s.e.m. (*n* = 3–7 mice per group).

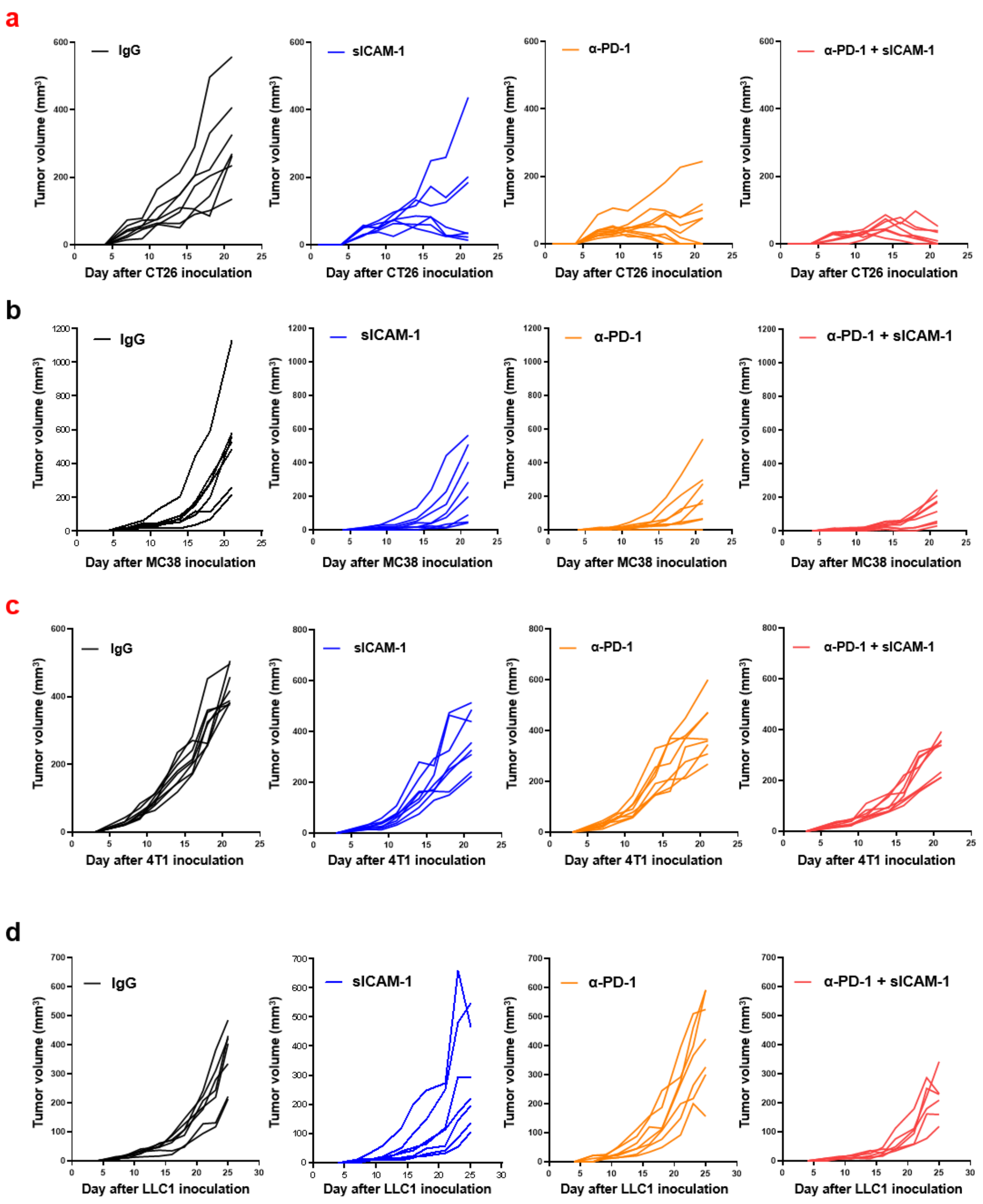

Supplementary Figure 11. Effect of sICAM-1 on tumor growth in mice treated with anti-PD-1. (**a**–**d**) Spider plot of CT26 (**a**), MC38 (**b**), 4T1 (**c**), and LLC1 (**d**) tumor growth illustrated in **Figure 5a**–**d**.

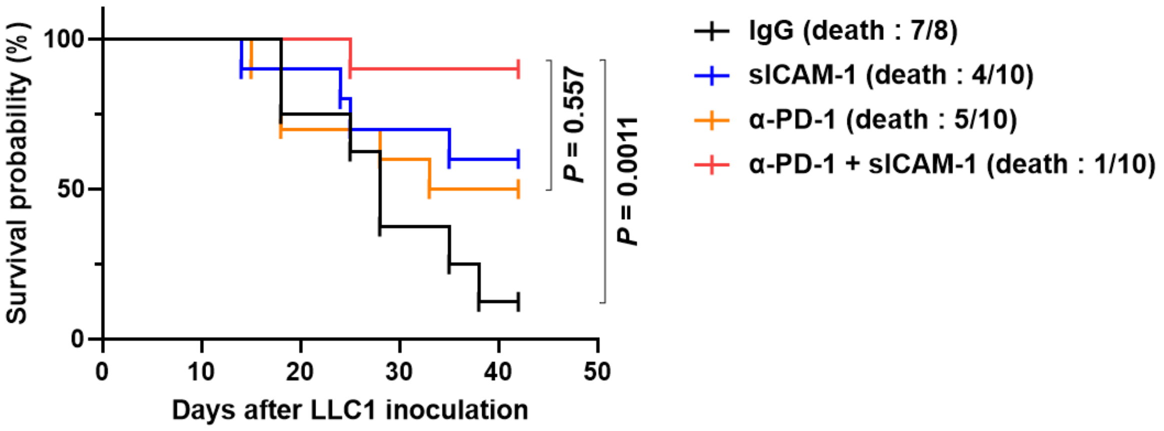

Supplementary Figure 12. The survival probability of mice with LLC1 treated with sIACM-1. The survival probability of C57BL/6 mice with orthotopic LLC1 treated with sICAM-1; anti-PD-1; or the combination. C57BL/6 mice were intercostally injected with LLC1 for the orthotopic model. A log-rank test was used for statistical analysis. (*n* = 8–10 mice per group)

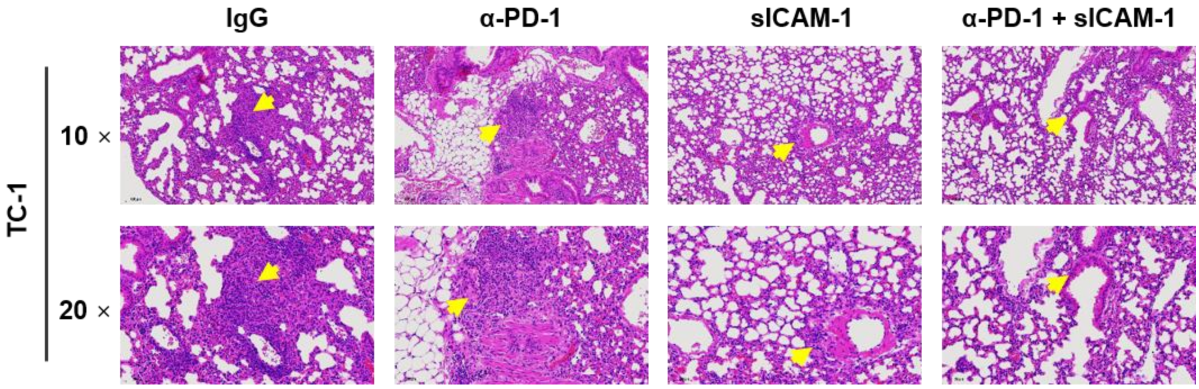

Supplementary Figure 13. The effects of sICAM-1 on TC-1 tumor. The images of H&E-stained tumor tissues from C57BL/6 mice with orthotopic TC-1 tumors treated with sICAM-1; anti-PD-1; or the combination at 10× or 20× magnifications. The mice assessed above was different from those in **Figure 5f**.

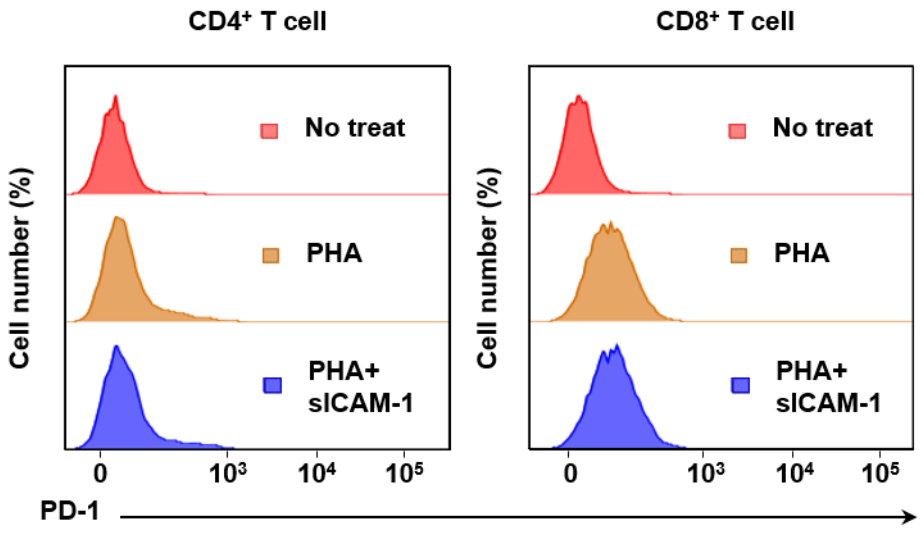

Supplementary Figure 14. The effect of sICAM-1 on exhausted T cells. PD-1 expression on $CD4^+$ and $CD8^+$ T cells treated with PHA-L or PHA-L + sICAM-1 using flow cytometry.
