# Supplementary material for: Intracellular Adhesion Molecule‐1 Improves Responsiveness to Immune Checkpoint Inhibitor by Activating CD8+ T Cells
Source: Adv Sci (Weinh). 2023 Apr 25;10(17):2204378. doi: 10.1002/advs.202204378 (PMC10265102; doi:10.1002/advs.202204378)
Supplement: Supplementary file 2 — Supporting Information [file ADVS-10-2204378-s003.pdf]

ADVANCED SCIENCE
Open Access

Supporting Information

for *Adv. Sci.*, DOI 10.1002/advs.202204378

Intracellular Adhesion Molecule-1 Improves Responsiveness to Immune Checkpoint Inhibitor by Activating $CD8^+$ T Cells

Se-Hoon Lee, Yeongmin Kim, Bu-Nam Jeon, Gihyeon Kim, Jinyoung Sohn, Youngmin Yoon, Sujeong Kim, Yunjae Kim, Hyemin Kim, Hongui Cha, Na-Eun Lee, Hyunsuk Yang, Joo-Yeon Chung, A-Reum Jeong, Yun Yeon Kim, Sang Gyun Kim, Yeonhee Seo, Sehhoon Park, Hyun Ae Jung, Jong-Mu Sun, Jin Seok Ahn, Myung-Ju Ahn, Hansoo Park and Kyoung Wan Yoon**

Intracellular adhesion molecule-1 improves responsiveness to immune checkpoint inhibitor by activating $CD8^+$ T cells

Supplementary Tables: pages 2-6

Supplementary Table 1 and 5 are Excel file types uploaded separately.

Supplementary Table 2. Baseline characteristics of the study population for survival analysis

		NSCLC patients (n = 84)
**Age**	**Median (Range)**	61.2 (20.9-82.6)
**Sex**		
	**Male**	60 (71.4%)
	**Female**	24 (28.6%)
**Smoking history**		
	**Never smoker**	31 (36.9%)
	**Ex-smoker**	17 (20.2%)
	**Current smoker**	35 (41.7%)
**Histology**		
	**Adenocarcinoma**	56 (66.7%)
	**Squamous-cell carcinoma**	23 (27.4%)
	**Other**	5 (5.9%)
**Molecular subtypes**		
	**Activating EGFR mutation**	8 (9.5%)
	**Activating KRAS mutation**	6 (7.1%)
**ECOG PS**		
	**0**	1 (1.2%)
	**1**	74 (88.1%)
	**2**	9 (10.7%)
**Previous RT history**		
	**n**	34 (40.5%)
**Backbone ICI**		
	**Pembrolizumab**	38 (45.2%)
	**Nivolumab**	31 (36.9%)
	**Atezolizumab**	6 (7.1%)
	**Durvalumab**	4 (4.8%)
	**Avelumab**	5 (6.0%)
	**Other**	0 (0.0%)
**ICI treatment line**		
	**1L**	11 (13.0%)
	**2L**	33 (39.3%)
	**≥3L**	39 (46.4%)
**Biopsy sites**		
	**Lung**	25 (29.7%)
	**Lymph node**	33 (39.3%)
	**Other**	26 (31.0%)

ECOG PS, Eastern cooperative oncology groups performance score; ICI, immune check-point inhibitor

Supplementary Table 3. Clinical outcomes of the study population for survival analysis

		**NSCLC patients (*n* = 84)**
**Overall response (*n*)**	**CR**	1 (1.2%)
	**PR**	26 (31.0%)
	**SD**	20 (23.8%)
	**PD**	37 (44.0%)
**Progression-free survival (days)**	**Median (95% CI)**	98.5 (177 – 302)
**Overall survival (days)**	**Median (95% CI)**	480.5 (487 – 661)

PR, partial response; SD, stable disease; PD, progressive disease

Supplementary Table 4. Clinical outcomes and survival analysis by cut-off using median TPM value of ICAM-1

NSCLC patients (*n* = 84)		Cutoff-by median TPM value of ICAM-1	
		**Above median (*n* = 42)**	**Below median (*n* = 42)**
**CR (*n*)**		0 (0.0%)	1 (2.4%)
**PR (*n*)**		17 (40.5%)	9 (21.4%)
**SD (*n*)**		12 (28.6%)	8 (19.0%)
**PD (*n*)**		13 (30.9%)	24 (57.1%)
**Median PFS (95% CI)**	*P* = 0.012*	154 (205-414)	65 (106-232)
**Median OS (95% CI)**	*P* = 0.070*	790.5 (567-824)	338.5 (345-560)

*P-value calculated by log-rank test

PFS and OS is described in days

PR, partial response; SD, stable disease; PD, progressive disease; TPM, transcripts per kilobase million; PFS, progression-free survival; OS, overall survival

Supplementary Table 6. Baseline characteristics of the study population for plasma analysis

		NSCLC patients (*n* = 77)
**Age**	**Median (Range)**	63.6 (32.8-82.6)
**Sex**		
	**Male**	70 (90.9%)
	**Female**	7 (9.1%)
**Smoking history**		
	**Never smoker**	8 (10.4%)
	**Ex-smoker**	40 (51.9%)
	**Current smoker**	29 (37.7%)
**Histology**		
	**Adenocarcinoma**	42 (54.5%)
	**Squamous-cell carcinoma**	27 (35.1%)
	**Other**	8 (10.4%)
**Molecular subtypes**		
	**Activating EGFR mutation**	7 (9.1%)
	**Activating KRAS mutation**	4 (5.2%)
**ECOG PS**		
	**0**	3 (3.9%)
	**1**	72 (93.5%)
	**2**	2 (2.6%)
**Backbone ICI**		
	**Pembrolizumab**	42 (54.5%)
	**Nivolumab**	9 (11.7%)
	**Atezolizumab**	26 (33.8%)

ECOG PS, Eastern cooperative oncology groups performance score; ICI, immune check-point inhibitor

Supplementary Table 7. Clinical outcomes of the study population for plasma analysis

		NSCLC patients (*n* = 77)
**Overall response (*n*)**	**PR**	30 (39.0%)
	**SD**	12 (15.6%)
	**PD**	35 (45.4%)

PR, partial response; SD, stable disease; PD, progressive disease
